# Structural insights and activating mutations in diverse pathologies define mechanisms of deregulation for phospholipase C gamma enzymes

**DOI:** 10.1016/j.ebiom.2019.102607

**Published:** 2020-01-06

**Authors:** Yang Liu, Tom D. Bunney, Sakshi Khosa, Kévin Macé, Katharina Beckenbauer, Trevor Askwith, Sarah Maslen, Christopher Stubbs, Taiana M. de Oliveira, Kasim Sader, Mark Skehel, Anne-Claude Gavin, Christopher Phillips, Matilda Katan

**Affiliations:** a Discovery Sciences, R&D, AstraZeneca, Cambridge, CB4 0WG, UK; b Institute of Structural and Molecular Biology, Division of Biosciences, University College London, Gower Street, London WC1E 6BT, UK; c Institute of Structural and Molecular Biology, Birkbeck College, Malet Street, London WC1E 7HX, UK; d European Molecular Biology Laboratory, Structural and Computational Biology Unit, Meyerhofstrasse 1, 69117 Heidelberg, Germany; e Drug Discovery Group, Translational Research Office, School of Pharmacy, University College London, 29-39 Brunswick Square, London, WC1N 1AX, UK; f MRC Laboratory of Molecular Biology, Cambridge CB2 0QH, UK; g Cambridge Cryo-EM Pharmaceutical Consortium, Thermo Fisher Scientific, 11 JJ Thomson Avenue, Madingley Road, Cambridge, CB3 0FF, UK; h Department for Cell Physiology and Metabolism, University of Geneva, Centre Medical Universitaire, Rue Michel-Servet 1, CH-1211 Geneva 4, Switzerland

**Keywords:** Disease-linked variants, Phospholipase C gamma, Structure, Mechanism

## Abstract

**Background:**

PLCγ enzymes are key nodes in cellular signal transduction and their mutated and rare variants have been recently implicated in development of a range of diseases with unmet need including cancer, complex immune disorders, inflammation and neurodegenerative diseases. However, molecular nature of activation and the impact and dysregulation mechanisms by mutations, remain unclear; both are critically dependent on comprehensive characterization of the intact PLCγ enzymes.

**Methods:**

For structural studies we applied cryo-EM, cross-linking mass spectrometry and hydrogen-deuterium exchange mass spectrometry. In parallel, we compiled mutations linked to main pathologies, established their distribution and assessed their impact in cells and *in vitro*.

**Findings:**

We define structure of a complex containing an intact, autoinhibited PLCγ1 and the intracellular part of FGFR1 and show that the interaction is centred on the nSH2 domain of PLCγ1. We define the architecture of PLCγ1 where an autoinhibitory interface involves the cSH2, spPH, TIM-barrel and C2 domains; this relative orientation occludes PLCγ1 access to its substrate. Based on this framework and functional characterization, the mechanism leading to an increase in PLCγ1 activity for the largest group of mutations is consistent with the major, direct impact on the autoinhibitory interface.

**Interpretation:**

We reveal features of PLCγ enzymes that are important for determining their activation status. Targeting such features, as an alternative to targeting the PLC active site that has so far not been achieved for any PLC, could provide new routes for clinical interventions related to various pathologies driven by PLCγ deregulation.

**Fund:**

CR UK, MRC and AstaZeneca.

**Research in context**

**Evidence before this study**

Extensive genetic evidence during last several years implicated PLCγ enzymes in disease development across diverse pathologies. However, only a few of the mutations have been assessed functionally. Furthermore, proposed mechanisms of regulation for PLCγ enzymes relied only on partial structural insights that generated conflicting data. Similarly, potential mechanisms for the impact of mutations have been suggested only for several initially discovered variants.

**Added value of this study**

We present here, the first structure of an intact, full length PLCγ obtained in a complex with the FGFR kinase domain, using cryo-EM. The PLCγ is in an auto-inhibited form and we define the main surfaces that contribute to auto-inhibition that, in turn, suggest physiological mechanisms of activation. We also, for the first time, compile mutations from different pathologies and perform comprehensive, comparative functional studies. Using the new structural framework, we define the major mechanistic class as changes impacting on the enzyme autoinhibition as well as other classes affecting different aspects of PLCγ regulation.

**Implications of all the available evidence**

The correlation of genetic data with the structural and mechanistic implications will facilitate their assessment in the clinical setting with respect to diagnosis and treatment. Furthermore, new structural insights and methods used to measure PLC activity, will directly facilitate on-going drug-discovery efforts based on rational design and other searches for new treatment options. This is particularly important because, so far, there are no suitable inhibitors (or modulators) for any of the PLC enzymes.

Alt-text: Unlabelled box

## 1 Introduction

External stimuli, such as neurotransmitters, agonists for immune cell receptors, hormones and growth factors, stimulate a range of different receptors that activate core signalling modules, including phospholipase C (PLC) enzymes that generate second messengers, diacylglycerol and inositol 1,4,5-trisphosphate [1], [2], [3]. Although PLC enzymes, and in particular the PLCγ family, have long been recognized as key components in this intracellular signal transmission, it is only during the last few years that their roles in disease development have become apparent. Crucially, a number of genetic studies revealed a PLCγ subnetwork as an important regulator of cell functions that can be subverted in various diseases that ultimately require new treatment options. Variants of the two family members (PLCγ1 and PLCγ2) have been linked to cancer, complex immune disorders, inflammation (further implicated in other diseases such as cancer and steroid-sensitive nephrotic syndrome) as well as Alzheimer's and related neurodegenerative diseases; the major, comprehensive discoveries are described in a number of publications including [4], [5], [6], [7], [8], [9], [10], [11], [12], [13]. Specifically, the somatic mutations in *PLCG1* gene have been linked to angiosarcoma and T-cell lymphomas while somatic mutations in *PLCG2* contribute to drug (Ibrutinib) resistance in chronic lymphocytic leukaemia (CLL). Furthermore, dominantly inherited *PLCG2* mutations have been documented to cause autoimmunity and inflammation (designated as PLAID for PLCγ2-associated antibody deficiency and immune dysregulation and as APLAID for autoinflammation, antibody deficiency, and immune dysregulation). *PLCG2* variants have been also associated with inflammatory bowel disease (IBD) and one rare *PLCG2* variant has been reported to strongly associate with the protection from the development of Alzheimer's disease (AD). Interestingly, in a number of cases where functional characterization of the effect of genetic changes on PLC activity has been performed, these alterations (predominantly single amino-acid substitutions) result in an increase of PLCγ activity [6,8,14,15].

Numerous studies identified various interacting proteins involved in cellular regulation of PLCγ enzymes [3,16]. In the immune cells, several interconnected adapter proteins (such as LAT, Gads and SLP76 in T-cells) are involved in positioning of PLCγ for further phosphorylation by non-receptor tyrosine kinases and for access to the membrane-bound substrate, phosphatidylinositol 4,5-bisphosphate [PtdIns(4,5)P_2_]. In contrast, receptor tyrosine kinases (RTKs) provide both the scaffold and the kinase activity that, *via* phosphorylation, triggers a conformational change to an active state of PLCγ. Both types of signalling connectivity are relevant for pathology; for example, in angiosarcoma, mutations in PLCγ1 are mutually exclusive with the activating mutations in the upstream RTK [17]. With respect to mechanistic aspects of PLCγ regulation, experimental evidence is most extensive for activation by one of the RTKs, namely the fibroblast growth factor receptor 1 (FGFR1), where the main site of binding on FGFR1 (pY766) and the key site of phosphorylation in PLCγ1 (pY783) are clearly defined [18], [19], [20]. However, the interaction surfaces between the FGFR1 and PLCγ1 and the precise mechanism of subsequent PLCγ1 activation remain controversial [18,20]. Nevertheless, the current model based on partial structural insights for this system and on studies of cellular signalling, outlines that autoinhibition defines the inactive state of PLCγ and that the release of these intramolecular inhibitory constrains provides the first step leading to activation [16]. Although some elements involved in the autoinhibition have been defined, the extent and nature of these interactions and whether and how they result in the occlusion of the active site have not been characterised. Interestingly, the initially reported disease-linked mutations have been suggested to impact on the autoinhibition [16]. However, further understanding of physiological activation, dysregulation by ever increasing number of mutations discovered in diverse pathologies and advances in drug discovery are all critically dependent on currently lacking structures of the intact PLCγ enzymes.

Here we combine several experimental approaches to characterize an intact PLCγ1 enzyme in its autoinhibited form and define interactions with FGFR1. By using this architectural framework and direct assessment of different PLCγ variants linked to diseases, we outline different mechanisms causing PLCγ dysfunction.

## 2 Materials and methods

### 2.1 Constructs, protein purification and protein complexes

Full-length human PLCγ1 containing a C-terminal Myc-tag or YFP-tag was cloned using Gateway technology (Thermo Fisher) into pDONR207 (Thermo Fisher) and after sequencing transferred by the LR reaction into a Gateway modified version of pTriEx4 (Novagen). Amino acid substitutions and deletions were prepared utilising the Quikchange II Site-Directed Mutagenesis Kit (Agilent) following manufacturer's instructions.

For expression, Freestyle 293F cells (RRID:CVCL_D603) were grown in suspension on a platform shaker in a humidified 37 °C CO_2_ incubator (Infors) with rotation at 130 rpm. Cells were maintained between 4 × 10^5^ and 3 × 10^6^ cells/ml in a volume of 350 ml in 1 L culture flasks using Freestyle 293F Expression Medium (Invitrogen). For transfection, 350 ml of 293F cells (1.0 × 10^6^ cells) were mixed with plasmid DNA:PEI complexes prepared as follows. 14 ml of OptiPRO SFM™ (Invitrogen) supplemented with 4 mM of l-Glutamine was mixed with 437.5 µg of DNA and a volume of PEI (~25 kDa branched) at 1 mg/ml that is 1.5 times the mass ratio of the amount of DNA. The transfection mix was incubated at room temperature for 15 min before being added to the 293F cells and incubated at 37 °C with shaking. Five mM of sodium butyrate was added to the flask 24 h post transfection. Following a total incubation of 48 h at 37 °C with shaking, the cells were pelleted by centrifugation at 300 × *g* for 15 min. The pellets were snap frozen in liquid nitrogen and then stored at −80 °C.

Cell pellets corresponding to 15 to 25 g of material were resuspended in 25 ml of 293F Lysis Buffer (25 mM Tris.Cl, 250 mM NaCl, 40 mM Imidazole, 10 mM Benzamidine, 1 EDTA free protease inhibitor tablet (Roche), pH 8.0). Cell lysis was performed using a sonicator probe supplying 5 × 30 s between each pulse while material was kept on ice. Lysed material was clarified by centrifugation at 4 °C for 2 h at 18,000 rpm in a Sorvall SS34 rotor.

The clarified sample was applied to a 5 ml HisTrap column (GE Healthcare) on an Akta Explorer system (GE Healthcare) utilizing His-Buffer A (25 mM Tris.Cl, 500 mM NaCl, 40 mM Imidazole, 1 mM TCEP, pH 8.0). Non- specifically bound proteins were removed by washing the column with 10 column volumes of His Buffer A. His-tagged recombinant proteins were eluted with a linear gradient from His Buffer A to His-Buffer B (25 mM Tris.Cl, 500 mM NaCl, 500 mM Imidazole, 1 mM TCEP, pH 8.0) over 5 column volumes. Fractions containing the recombinant protein were pooled and then applied to a Superdex 200 26/60 column (GE Healthcare) equlilibrated in Gel Filtration buffer A (25 mM HEPES, 150 mM NaCl, 2 mM TCEP, pH 7.5). Fractions were collected, pooled and concentrated in Vivascience spin concentrators (Vivaproducts), snap frozen in liquid nitrogen and stored at −80 °C.

A synthetic ORF encoding for the human FGFR1 kinase domain (456–774, L457V, Y463F, C488A, Y583F, C584S, Y585F, Y653F and Y654F) has been described earlier [19] was cloned into the plasmid pOPINS.

Constructs were transformed into *E.coli* strain C41 (DE3). Colonies were inoculated into 500 ml of terrific broth and grown to an OD600 of 1.0. Cultures were cooled to 15 °C and expression was induced with 100 μM IPTG for approximately 16 h. Bacteria were pelleted and stored at −20 °C until processed.

Bacterial pellets were resuspended in 20 ml of chilled Lysis Buffer (25 mM Tris.Cl, 250 mM NaCl, 40 mM Imidazole, 10 mM Benzamidine, 1 mM MgCl_2_ and 100 μM CaCl_2_, 100 μg/ml lysozyme, pH 8.0). Resuspension was accomplished by placing the pellets on an orbital shaker set at 200 rpm at 4 °C for 30 min. Lysis was continued by the addition of 5 ml of a solution of 10% (v/v) Triton-X-100 and 1 Kunit of bovine pancreatic DNAse I, on the orbital shaker at 200 r.p.m. at 4 °C for 1 h. Clarification of the lysate was performed by centrifugation of the sample for 1 h at 4 °C at 18,000 rpm in an SS34 rotor (Sorvall). The clarified sample was applied to a 5 ml HisTrap column (GE Healthcare) on an Akta Explorer system (GE Healthcare) utilizing His-Buffer A (25 mM Tris.Cl, 500 mM NaCl, 40 mM Imidazole, 1 mM TCEP, pH 8.0). Non- specifically bound proteins were removed by washing the column with 10 column volumes of His Buffer A. His-tagged recombinant proteins were eluted with a linear gradient from His Buffer A to His Buffer B (25 mM Tris.Cl, 500 mM NaCl, 500 mM Imidazole, 1 mM TCEP, pH 8.0) over 5 column volumes. Eluted protein was quantified using a Nanodrop (Thermo Scientific) using the extinction coefficient of the protein. The N-terminal tags on the proteins were cleaved overnight by addition of Ulp1 protease.

The protein/protease mix was dialysed overnight at 4 °C against 500 ml of Dialysis Buffer (25 mM Tris.Cl, 150 mM NaCl, 10 mM Imidazole and 1 mM TCEP, pH 8.0). Subsequently, proteins were passed again over the 5 ml HisTrap column and material that did not bind was collected. These proteins were dialysed against Low Salt Dialysis Buffer (25 mM Tris.Cl, 20 mM NaCl, 1 mM TCEP, pH 8.0) for a minimum of 3 h at 4 °C. Subsequently, proteins were further purified by application to a 5 ml HiTrap Q column (GE Healthcare) in Q Buffer A (25 mM Tris.Cl, 20 mM NaCl, 1 mM TCEP, pH 8.0) and eluted in a linear gradient to 50% of Q Buffer B (25 mM Tris.Cl, 1 M NaCl, 1 mM TCEP, pH 8.0) over 25 column volumes. Fractions containing the recombinant protein were pooled and then applied to a Superdex 75 26/60 column (GE Healthcare) equlilibrated in Gel Filtration Buffer B (25 mM Tris.Cl, 150 mM NaCl, 1 mM TCEP, pH 8.0). Fractions were collected, pooled and concentrated in Vivascience spin concentrators (Vivaproducts), snap frozen in liquid nitrogen and stored at −80 °C.

Purified hPLCγ1 (or mutant) protein was mixed with purified FGFR1-1P protein in a molar ratio of 1:10, incubated on ice for 30 min and applied to a Superdex 200 26/60 column (GE Healthcare) previously equilibrated in Gel Filtration Buffer A. Fractions corresponding to the protein complex were collected, pooled and concentrated in Vivascience spin concentrators (Vivaproducts). Fractions corresponding to excess FGFR1-1P were pooled and concentrated separately. Twenty mM of BS3 *crosslinker* (ThermoFisher Scientific) was added to 4 mg of concentrated protein complex and 500 µl of Gel Filtration buffer A containing 0.33 mg excess FGFRint and incubated at room temperature for 1 hr. Fifty µl of 1 M Tris pH 8.0 was added to the above mixture and further incubated for 15 min. The complex mixture was then reapplied to a Superdex 200 26/60 column (GE Healthcare) equilibrated with Gel Filtration buffer A. The elution peak fractions corresponding to crosslinked protein complex were collected, pooled and concentrated in Vivascience spin concentrators (Vivaproducts). The crosslinked protein complex was then applied to a Superdex 200 10/300 GL column (GE Healthcare) previously equilibrated with Gel Filtration buffer A. The elution peak fractions corresponding to crosslinked complex of FGFR1-1P and hPLCγ1 (or mutant) were collected, their concentrations were measured, snap frozen in liquid nitrogen and stored at −80 °C.

For generation of lentiviruses, full-length human PLCγ1 was cloned into a modified pLEX_307 plasmid (Addgene) for subsequent transduction into Human Umbilical Vein Endothelial Cells (HUVEC) cells grown in EGM-2MV medium (Lonza). PLCγ1 was expressed in the presence of Doxycycline (1 μg/ml) and, 9 days after seeding, cell morphology was analysed using a Zeiss AxioImager A1.

### 2.2 Measurements of PLC activity in cells

For the measurements of PLC activity, COS-7 cells (RRID:CVCL_0224) were cultured in DMEM (Sigma) containing 10% (v/v) FBS and 2.5 mM glutamine (growth media). Cells were grown as a monolayer at 37 °C in 5% CO_2_. COS-7 cells were seeded into 96-well plates at a density of 7500 cells per well in 0.1 mL of growth media and incubated overnight. Fresh media was applied and the cells transfected with plasmid DNA at 100 ng/well that had been diluted in 5 μl jetPRIME® buffer and 0.2 μl jetPRIME® (Polyplus) that was prepared as instructed by the manufacturer. The DNA concentration was kept constant by adding empty plasmid. Each PLCγ1 construct was transfected at 4 concentrations in triplicate as outlined in the figures. Twenty-four hours post transfection, the media was removed and replaced with growth media without FBS but containing 0.25%(w/v) fatty acid free BSA. The COS-7 were then incubated for a further 24 h. Subsequently, the media was replaced with growth media without FBS but containing 50 mM LiCl with and without 100 ng/mL EGF and incubated for a further 1 h. The media was aspirated and replaced by 25 μl of Stimulation Buffer (20 mM HEPES.OH, 2 mM CaCl_2_, 1 mM MgCl_2_, 8.4 mM KCl, 292 mM NaCl, 11 mM glucose and 100 mM LiCl, pH 7.4) followed by 25 μl of lysis buffer (50 mM HEPES.OH, 0.8 M KF, 0.2%(w/v) BSA and 1% (v/v) Triton-X-100, pH 7.0). The cells were lysed for 30 min at room temperature on an orbital shaker. Seven microlitres of the cell lysate was pipetted in duplicate into a white 384 well plate (Greiner Bio-One) followed by 1.6 μl of IP1-d2 (Cisbio). After 5 min, 1.6 μl of anti-IP1-Cryptate (Cisbio) was added and the plate sealed and incubated at room temperature for 1 h. The plate was read on a PHERAstar (BMG Labtech) plate reader in HTRF mode and the data converted to IP_1_ concentration using a standard curve generated following manufacturer's instructions.

Quantities of expressed proteins were measured using a WES Western Blotting system (Protein Simple). For this, a further 96-well plate was transfected identically to the plate used in the IP_1_ assay described above. Forty-eight hours post transfection the cells were washed once in ice-cold PBS and subsequently lysed in 50 μl RIPA buffer (Thermo Fisher) containing a protease and phosphatase inhibitor cocktail (Thermo Fisher). The cells were freeze-thawed at −20 °C and subsequently 4 μl of each well loaded on a WES Western Blotting. Proteins were detected with a 1:150 dilution of the anti-PLCγ1 05-163 (Millipore, RRID:AB_309638)) and a 1:150 dilution of the anti- β-actin antibody 13E5 (Cell signaling Technology, RRID:AB2223172).

Inositol phosphate formation in transfected COS7 cells incubated in the medium containing myo-[2-3H] inositol (MP Bio- 253medicals) was assessed as described in Everett et al. [21].

### 2.3 Quantification of enzyme activity of PLCγ1 variants *in vitro*

Determination of PLC activity *in vitro* was performed by two methods. Firstly, the PLC driven hydrolysis of phosphatidylinositol in mixed micelles was monitored by quantifying the production of inositol phosphate (IP_1_) using the IPone kit (CisBio) in an endpoint assay format. Secondly, the real time hydrolysis of the synthetic substrate PLCglow (KXTbio) was monitored in a continuous assay format. Essentially both methods utilised the following Assay Buffer (50 mM HEPES.OH, 70 mM KCl, 3 mM EGTA, 2.9 mM CaCl_2_, 2 mM TCEP, 50 μg/mL fatty acid free, pH 7.0).

For the measurement of IP_1_ production, the lipid substrate phosphatidylinositol was dried under vacuum in a glass test tube and resuspended in Assay Buffer containing 0.6%(w/v) sodium cholate to give a final lipid concentration of 100 μM. The PLC proteins were assayed at a final concentration of 60 nM (full length variants) or 25 nM (ΔSA variants) in a total assay volume of 200 μL. Samples of 10 μL were removed at the times given in the text and the reaction stopped by the addition of 20 μL of 50 mM EGTA, pH 8.0. The amount of IP_1_ was quantified through the addition of a labelled IP_1_ probe and an anti-IP_1_ labelled cryptate antibody and monitored by HTRF as outlined in the manufacturer's instructions. The amount of IP_1_ produced was calculated by interpolation from a standard curve.

In the continuous assay format the enzymatic activity of PLC variants were quantified using the soluble PLCglow substrate. Activity was measured in low volume black 384 well plates. 20 pM PLC proteins were combined with a serial dilution of PLCglow substrate to a final volume of 10 μl. Cleavage of PLCglow by PLC enzymes releases a fluorescent 6-aminoquinoline which was measured with a Clariostar multimode plate reader (BMG Labtech Bmh) with an excitation wavelength of 344 nm, emission wavelength of 540 nm. Initial velocities were calculated for several substrate concentrations for each PLC variant and the Michaelis-Menten kinetic parameters were calculated.

### 2.4 Statistical analysis

For PLC activity measurements in cells, experimental duplicates of biological triplicates were presented as the mean and SEM. For *in vitro* PLC activity measurements, each data point was collected in triplicate and kinetic parameters presented as the mean and SEM.

### 2.5 Thermofluor assay

A PCR microplate was filled with 20 μL protein solution (1 mg/ml) of cSH2 variant in Thermofluor buffer (25 mM Tris.Cl, 150 mM NaCl, 1 mM TCEP, pH 8.0). Five μL of SYPRO Orange dye (ThermoFisher) was added to give a final dye dilution of 500 times the stock and a final sample volume of 25 μL. The microplates were sealed with an adhesive optical clear seal. Samples were heated using a RT-PCR instrument (iQ5, BioRad) and the increase in fluorescence monitored from 10 °C to 90 °C in 1 °C increments. The temperature at which 50% of the protein is unfolded and bound to the fluorescent dye (apparent melting temperature, Tm) corresponds to the inflexion point of the slope. Raw data were plotted and analyzed using GraphPad Prism (http://www.graphpad.com). The transition midpoint was calculated automatically using an in-house written script. For each protein sample three to four aliquots were analyzed in the same experiment and data presented as the means +/- the SD.

### 2.6 LiMA

Recruitment of PLCγ1 variants to membranes was studied by LiMA as described previously [22]. Binding to phosphatidylinositol phosphates was probed against giant unilamellar liposomes (GUVs) formed from lipid mixtures containing individual PIP species (PI(3)P, PI(4)P, PI(5)P, PI(3,4)P_2_, PI(3,5)P_2_, PI(4,5)P_2_, PI(3,4,5)P_3_) at 10 mol% in a DOPC background. Cooperativity was probed by including an additional 10 mol% of PS into the mixtures containing PIPs in a DOPC background. GUVs formed from DOPC only or from DOPC with 10 mol% PS were included as controls in the array design. Liposomes were formed by rehydration of the chip with a physiological buffer (gel filtration buffer A) for 10 min. PLCγ1 variants purified as YFP-fusions from HEK cells were diluted to 0.2 mg/ml in the assay buffer and protein binding to GUVs was allowed for 60 min. After, unbound proteins were washed off by injection of 80 μl of assay buffer and automated epifluorescence microscopy was performed on a scanR High-content Screening Station (Olympus). For an additional readout of the enzymatic activity of the PLCγ1 variants, a consecutive incubation of the chip with the PH domain of PLCδ1 [23] was performed. sfGFP-tagged PLCδ1PH was used from an *E.coli* overexpression lysate. To obtain this lysate, a 100 μl *E. coli* overexpression cell pellet (corresponding to about 5 ml of culture) was resuspended in 1 ml of lysis buffer (10 mM Hepes, pH 7.5, 150 mM NaCl, 0.5 mM DTT, Benzonase nuclease (Millipore), 1x of each Pefabloc (Sigma-Aldrich), E-64 (Sigma-Aldrich), Aprotinin A (Sigma-Aldrich), Pepstatin (Sigma-Aldrich), Bestatin (Sigma-Aldrich), Leupeptin (Sigma-Aldrich)) and sonicated using a probe tip sonicator (Branson Digital Sonifier) on ice (total 1 min, ON 0.5 s, OFF 10 s, amplitude 15%). The cell lysate was centrifuged at top speed for 30 min at 4 °C in an Eppendorf 5424R centrifuge to remove cell debris and protein aggregates. Protein integrity in the cell lysate was verified by SDS-PAGE and Western blotting with an anti-GFP antibody (Miltenyi Biotec). Protein concentration in the cell lysate was adjusted to a fluorescence intensity of 400 rfu using a microplate reader (Synergy 4, BioTek). Binding to GUVs was allowed for 30 min followed by washing with assay buffer and re-imaging of the liposome microarray chip. A normalized binding intensity (NBI) was obtained for each protein-lipid combination tested as described previously [22]. NBIs were further normalized to the median NBI of the DOPC spots distributed across each LiMA array. As most proteins do not specifically bind to DOPC, the binding to DOPC can be used to correct for background signal. Background corrected NBIs were further min max normalized and a mean binding intensity of >3 replicates was calculated. Binding data was represented in a heatmap. To obtain the activity value, the reciprocal of the NBI of PLCδ1PH binding to GUVs formed from 10 mol% PI(4,5)P_2_ + 10 mol% PS in DOPC was calculated. Activity values of the PLCγ1 variants were set relative to the WT activity and mean values with SD were calculated from >3 replicates.

### 2.7 Mass spectrometry

*Hydrogen deuterium exchange mass spectrometry (HDX-MS)*- Protein stock solutions (apo PLCγ1, apo FGFR1, PLCγ1:FGFR1 complex from SEC) of 10 μM in 20 mM Tris-HCl pH 7.8, 25 mM NaCl, 10 mM MgCl_2_ and 1 mM TCEP were used in the HDX-MS experiments. These samples were kept at 1 °C. Liquid handling was performed using a LEAP PAL RTC (LEAP Technologies) and the HDX workflow (digestion, separation) performed on a Waters HDX Manager (Waters).

Deuterium exchange reactions were initiated by diluting the proteins 1:20 into labelling buffer (20 mM Tris-HCl pH 7.8, 25 mM NaCl, 10 mM MgCl_2_, 1 mM TCEP in D_2_O) giving a final D_2_O concentration of 92% (v/v). Labelling experiments were performed as technical duplicates at 5 different time points: 30 s, 60 s, 300 s, 900 s, 1800s, 3600 s. The labelling reactions were quenched by 1:1 dilution into 1.6% (v/v) formic acid in 3 M urea at 1 °C. Samples were immediately processed after quenching.

Quenched samples (7.5 pmol) were immediately injected onto an Enzymate BEH immobilised pepsin column (2.1 × 300 mm, 3 μm; Waters) at 100 μL/min at 20 °C for 3 min at 10,000 psi. the peptides were trapped and desalted on an Acquity BEH C18 VanGuard pre-column (130 Å, 2.1 × 5 mm, 1.7 μm; Waters) kept at 0.1 °C. Peptides were eluted using a 6 min gradient of 5–35% acetonitrile in 0.1% (v/v) formic acid at 40 μL/min on an Acquity UPLC BEH C18 column (130 Å, 1.7 μm, 1 mm × 100 mm; Waters) at 0.1 °C. Peptides were detected on a SYNAPT G2-Si HDMS mass spectrometer (Waters) acquiring over an *m/z* range of 50–2000 with an electrospray source and lock mass calibration (Leucine Enkephalin, 200 pg/μL; Waters). The mass spectrometer was operated at a source temperature of 80 °C and a spray voltage of 2.5 kV. Spectra were collected in positive ion resolution mode.

Peptide identification was performed in Protein Lynx Global Server (Waters) using MS^E^ data collected for undeuterated control samples. The peptide lists were imported into DynamX (Waters) where peptides were filtered: minimum intensity of 10,000, minimum of 0.3 products per amino acid, a maximum MH+ error of 5 ppm, and identified in at least 50% of the undeuterated datasets. Automatic peptide assignment in DynamX was performed using the standard parameters, but all assignments were manually inspected, and ambiguous/erroneous assignments were excluded. Data were not corrected for back-exchange.

The curated deuterium uptake data were further processed using an in-house MATLAB script. Fractional deuterium uptake values were calculated as the measured deuterium uptake divided by the number of exchangeable backbone amides (excluding prolines and the first two residues). Peptide-level differences between the apo/complex states were then calculated as below, where differences ≥ 0.5 Da were considered significant.

$${\text{pepdiff}}_{i}^{\text{complex}}{\;=\;\text{uptake}}_{i}^{\text{complex}}-{\text{uptake}}_{i}^{\text{apo}}$$

**pepdiff_i_^co^^m^^plex^**: Difference (Da) in deuterium uptake for peptide **i** in state of interest; **uptake_i_^complex^**: Uptake (Da) for peptide **i** in state of interest; **uptake_i_^apo^**: Uptake (Da) for peptide **i** apo state.

The residue level differences were approximated according to the equation below, which averages the differences in deuterium uptake for a residue across all peptides in which it appears, normalised for the number of exchangeable residues in the peptides.

$$\text{resdif}f_{j}\;=\;\frac{1}{N}\sum_{i=1}^{N}\frac{\text{pepdif}f_{i}}{\text{amide}s_{i}}$$

**resdiff_j_**: mean difference for residue **j; N**: number of peptides containing residue **j; pepdiff_i_**: deuterium uptake difference for peptide **i** that contains residue **j; amides_i_**: number of exchangeable residues within peptide **i**.

*Cross-linking mass spectrometry (XL-MS)*-The purified PLCγ1/FGFR1 complex (25 mM HEPES pH 7.5, 150 mM sodium chloride, 2 mM TCEP) was cross-linked with a 100-fold excess of the N-hydroxysuccinimide (NHS) ester bis(sulfosuccinimidyl)suberate (BS3, ThermoScientific, USA), with respect to the protein concentration. The cross-linking reactions were incubated for 60 min at room temperature and then quenched by the addition of NH_4_HCO_3_ to a final concentration of 20 mM and incubated for further 15 min.

The cross-linked proteins were reduced with 10 mM DTT and alkylated with 50 mM iodoacetamide. Following alkylation, the proteins were digested with trypsin (Promega, UK) at an enzyme-to-substrate ratio of 1:100, for 1 h at room temperature and then further digested overnight at 37 °C following a subsequent addition of trypsin at a ratio of 1:20.

Peptides were analyzed by nano-scale capillary LC-MS/MS using an Ultimate U3000 HPLC (ThermoScientific Dionex, USA) to deliver a flow of approximately 300 nl/min. A C18 Acclaim PepMap100 5 μm, 100 μm × 20 mm nanoViper (ThermoScientific Dionex, USA), trapped the peptides before separation on a C18 Acclaim PepMap100 3 μm, 75 μm × 250 mm nanoViper (ThermoScientific Dionex, USA). Peptides were eluted with a gradient of acetonitrile. The analytical column outlet was directly interfaced via a nano-flow electrospray ionisation source, with a quadrupole Orbitrap mass spectrometer (Q-Exactive HFX, ThermoScientific, USA). MS data were acquired in data-dependent mode using a top 10 method, where ions with a precursor charge state of 1+ and 2+ were excluded. High-resolution full scans (*R* = 120 000, *m/z* 300–1800) were recorded in the Orbitrap followed by higher energy collision dissociation (HCD) (26% Normalized Collision Energy) of the 10 most intense MS peaks. The fragment ion spectra were acquired at a resolution of 50 000 and dynamic exclusion window of 20 s was applied.

For data analysis, Xcalibur raw files were converted into the MGF format using Proteome Discoverer 2.1 (ThermoScientific, USA) and used directly as input files for MeroX [24]. Searches were performed against an *ad hoc* protein database containing the sequences of the proteins in the complex and a set of randomized decoy sequences generated by the software. The following parameters were applied for the searches: maximum number of missed cleavages 3; targeted residues K, S, Y and T; minimum peptide length 5 amino acids; variable modifications: carbamidomethylation of cysteine, oxidation of methionine; MS1 accuracy 5 ppm and 10 ppm for MS2; False Discovery Rate cut-off: 5%. Finally, each fragmentation spectrum was manually inspected and validated.

### 2.8 Cryo-EM

*Cryo-grid preparation-* Cryo-EM grids were prepared by applying 2.5 µl of cross-linked PLCγ1-FGFR1 protein complex (at a protein concentration of 0.6 mg/ml) on glow-discharged holey carbon EM grids (Quantifoil R1.2/1.3 300mesh, Großlöbichau, Germany) using a FEI Vitrobot Mark IV plunge-freezing device at 4 °C with 85% humidity.

*EM data acquisition*- Automated data collection was performed on a Titan Krios electron microscope (FEI) operated at 300 kV using a Falcon III detector in electron counting mode and a Volta phase plate using EPU software (FEI). Data were collected in four independent sessions with the same settings to give a total of 2191 movies. Each micrograph was collected as 75 movie frames at a dose rate of 0.7 e^−^/pixel/s for 60 s, with a total dose of ~37 e^−^ Å^−2^ The calibrated magnification was 75,000x, corresponding to a magnified pixel size of 1.07 Å/pixel.

*Data processing*- RELION-3.0 [25] was used for most image-processing steps. Micrographs were subjected to motion correction with MotionCor2 [26]. The contrast transfer function parameters were determined with GCTF1.18 [27]. Initial auto-picking was performed with a Gaussian blob as a template (Fernandez-Leiro and Scheres, 2017) using only a subset of the data set, and particles were extracted in a box of 160 pixels. Reference-free 2D classification yielded initial 2D classes that were subsequently used as references (a low-pass filter of 20 Å was applied to avoid reference bias) for automatic picking of the whole data set. In total, 498,000 particles were picked up from 2191 micrographs. Elimination of bad particles was performed over several rounds of reference-free 2D classification. 8000 random good quality particles were selected for *ab initio* model generation using the Stochastic Deepest Descent (SDG) algorithm in RELION-3.0. The resulting model was applied as an input for 3D classification and 3D auto-refinement. After two rounds of 3D classification, 155,175 particles from a single class were subjected to 3D auto-refinement in RELION-3.0. The final model contained 155,175 particles and reached an overall, estimated resolution of 5–8 Å. Local resolution was calculated with RESMAP within RELION.

The 3D cryo-EM map has been deposited to Electron Microscopy Data Bank with the accession code: EMD-10288.

*Model building*- For model building, domains/arrays from 3 crystal structures (PDB 4QJ3, 2FJL and 3GQI) were first docked on the entire map by rigid body using CHIMERA (RRID:SCR_004097). The map was segmented domain by domain using the “zone” tool in CHIMERA (distance of 5 Å). The docking of each domain was then optimised by rigid body fitting into the segmented maps obtained. Essentially the same model was obtained when using PLCγ1 core homology model instead of 4QJ3.

Subsequent analyses, based on the docked structures, included the use of Phyre2 (https://www.ncbi.nlm.nih.gov/pubmed/25950237) and iTasser (https://www.ncbi.nlm.nih.gov/pubmed/20360767). The XL-MS data based on an acceptable distance constraint of about 20–30 Å between C_α_ atoms were also included. A number of crosslinks involve flexible/linker sequences not presented in the model and consequently, although potentially consistent, have not been included. The built models were further assessed using Phenix (https://scripts.iucr.org/cgi-bin/paper?dz5186,RRID:SCR_014224) and the highest scoring model amongst them was used.

## 3 Results

### 3.1 The architecture of PLCγ1 and of its complex with FGFR1

**Fig. 1:**
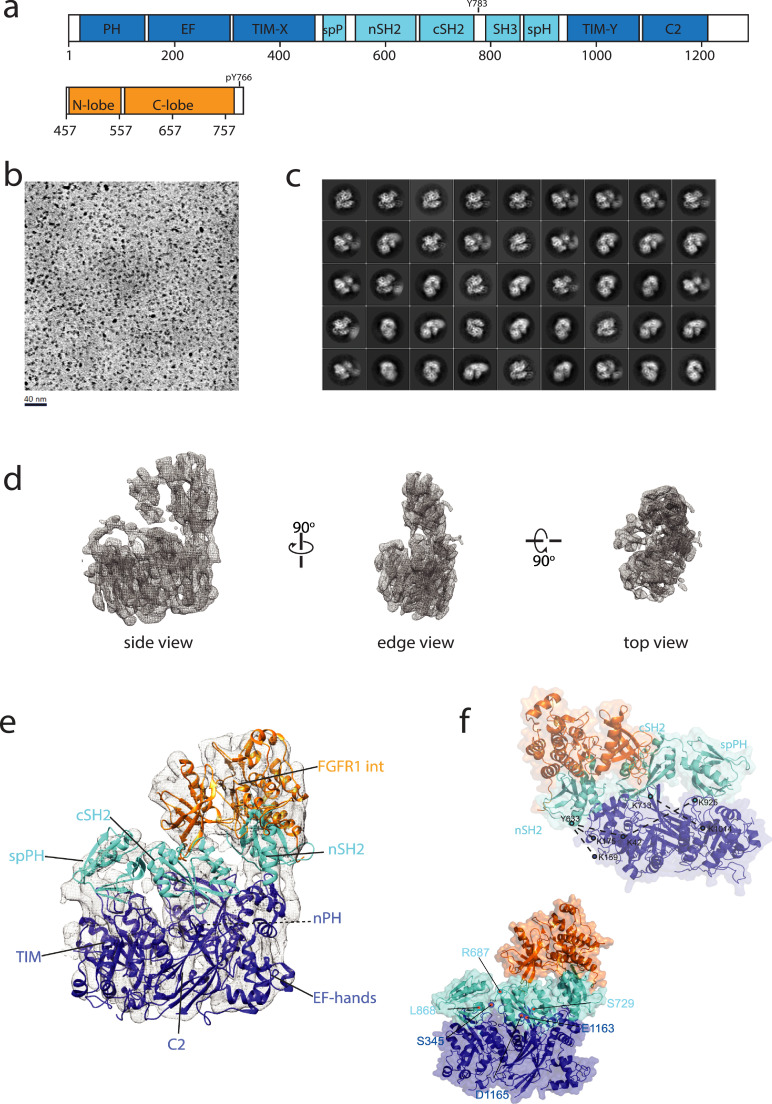
Complex of PLCγ1 and the intracellular part of FGFR1. (a) Domain organisation of PLCγ1 and FGFR1 intracellular region (FGFRint). The PLC-core domains (nPH, EF-hands, TIM-barrel and C2 domain) and regulatory domains unique for PLCγ (spPH, nSH2, cSH2 and SH3, comprising a γ-specific array - γSA) are coloured dark and light blue, respectively. The FGFRint kinase domain is coloured orange. (b) Representative cryoEM micrograph collected on a Krios microscope fitted with a Volta phase plate and Falcon 3 detector. (c) Representative 2D classes generated with Relion. (d) Different views of the 3D map presented as mesh. (e) Architecture of the PLCγ1/FGFR1int complex with individual domains docked into the 3D map. (f) Model of the complex with the domains represented as cartoon and surface. The orientation of the model where the crosslinks (black dotted lines) between the domains in the PLC-core (dark blue) and the regulatory region (light blue) can be visualised is shown at the top. All crosslinks are within the restraints described in (Bullock et al., 2018). The model presented in the same orientation as in (e) is shown at the bottom. The positions of the most frequent PLCγ mutations discovered in different pathologies, are indicated using PLCγ1 numbering as red dots. Residues R687, L868 and S729 correspond to R665, L845 and S707 in PLCγ2 and are the most frequently mutated in the latter isoform. (For interpretation of the references to color in this figure legend, the reader is referred to the web version of this article.)

As we described previously, the complex between the intracellular portion of FGFR1 (FGFR1int) and an intact PLCγ1 is very strong (K_D_ = 5 nM) [19] and can be assembled *in vitro* and purified (Supplementary Figure S1a). The conditions used for complex formation, *i. e*. the absence of ATP and therefore no phosphorylation, are not compatible with PLCγ1 activation and the enzyme is likely to be predominantly in its autoinhibited form. To obtain the structure of this complex, the protein preparations were subjected to mild crosslinking and further purification. Cryo-EM was performed using a Krios microscope fitted with a Volta phase plate and a Falcon III detector (Fig. 1). The representative 2D classes and the 3D map corresponding to the most abundant 3D class are shown in Fig. 1c and d. Calculated local resolutions for this map show a range of about 5–8 Å (Supplementary Figure S1). For PLCγ1 enzyme the structures for all isolated regulatory domains have been determined by X-ray crystallography or NMR (2FJL, 4FBN, 4EY0 and 1Y0M) including complexes with FGFRint (3GQI and 5EG3). The domains comprising the PLC-core and their relative orientations in other PLC families are well conserved (4QJ3, 2ISD and 2ZKM) and were used to generate a homology model for the PLC-core of PLCγ1. All these structurally defined fragments present in the PLCγ1/FGFRint complex, except the SH3 domain, could be docked into the 3D map of intermediate resolution (Fig. 1e and Supplementary Figure S2). The rigid body fitting of each individual domain has an expected cross correlation value (Supplementary Figure S2) and the model is also consistent with the known relative orientations of the domains in both, the PLC-core (4QJ3, 2ISD and 2ZKM) and the regulatory region (3GQI and 4EY0) as well as with the lengths of relevant linkers [19]. The resulting model is further supported by the HDX-MS and XL-MS data. Fig. 1f (top) illustrates the crosslinks between the regulatory and core domains of PLCγ1 while the interaction between FGFR1int and PLCγ1 is described further in a separate result section (see below).

The absence of density in the 3D map that could accommodate the SH3 domain is most likely caused by the flexibility of relative positions of this domain towards the rest of the molecule. All other domains from the regulatory region form an extensive interface with the PLC-core with the spPH domain facing the loop region on the TIM barrel, the cSH2 domain is proximal to the loop region on the C2 domain and the nSH2 domain is close to the EF-hands (Fig. 1e and Supplementary Figure S2). In this arrangement, the spPH domain occludes the loop region close to the active site opening on the TIM-barrel. Similarly, the canonical phosphopeptide binding site in the cSH2 domain is not accessible and positioned towards the C2 domain. In contrast, the phosphopeptide binding site in the nSH2 domain is in the right orientation to bind pY766 in the C-terminal tail of FGFR1. As described in detail further below, the relative orientation of the FGFR1int and the SH2 tandem in the context of the intact PLCγ1 is consistent with one of the previously reported partial structures containing FGFR1int (3GQI); our data further extend the understanding of these interactions.

Overall, these new structural insights define the auto-inhibited form of PLCγ1, stabilized by the extensive interactions between the PLC-core and regulatory domains, and the interaction with FGFR1 centred on the nSH2 domain.

### 3.2 Distribution and functional impact of mutations in PLCγ enzymes

Mutations affected either PLCγ1 or PLCγ2 across the spectrum of main pathologies were collected from available sources (Supplementary Figure S3 and Supplementary References). Analyses of distribution of these mutations show clustering of mutations to four main regions (Supplementary Figure S3). Firstly, TIM-barrel surfaces forming the active site opening, mapping to the loop regions (L1 and L3). Secondly, surfaces on the C2 domain formed by the loop regions, in particular the CBR3 loop and also part of cβ5. Thirdly, the cSH2 domain, most notably βF with surrounding loops and αA-βB and αB-βG loops. Fourthly, a region in spPH (β2 and β3) and, in particular, the proximal loop linking the SH3 domain to the second half of the spPH domain. These four regions (Supplementary Figure S3) overlap with the surfaces that are implicated in autoinhibition of the enzyme (Fig. 1E and Supplementary Figure S2). Five of the most frequent mutations in PLCγ enzymes are within the two proximity areas, spPH/TIM barrel and cSH2/C2 domain (Fig. 1f, bottom and Supplementary Figure S2e). The fact that no mutations have been reported in the regions of the nSH2 domain and the EF-hands close to each other, might suggest that they are not likely to be engaged in autoinhibitory interactions.

**Fig. 2:**
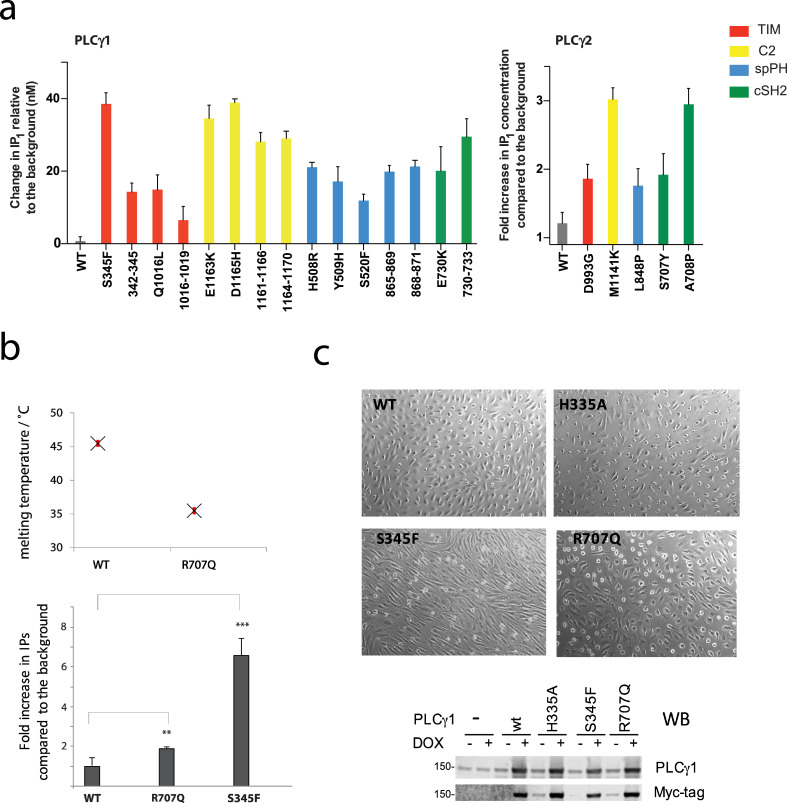
Functional assessment of PLCγ variants in a cellular context. (a) PLC activity in transfected COS-7 cells using the IP_1_ assay for the indicated variants of PLCγ1 (left) and PLCγ2 (right). For PLCγ1, the IP_1_ concentrations are calculated from the difference between the experimental condition and mock transfected cells and represent biological triplicates of experimental duplicates. For PLCγ2, the data are calculated from the fold increase in IP_1_ between the experimental condition and mock transfected cells and represent biological duplicates. The values are presented as the mean and SEM. (b) Characterisation of PLCγ1 variants reported in angiosarcoma. Melting temperature for the isolated cSH2 domains, WT and R707Q substitution calculated using the thermofluor assay (top). Basal PLC activity of PLCγ1 WT, R707Q and S345F variants in transfected COS-7 cells (bottom). (c) Morphology of HUVEC cells expressing the indicated PLCγ1 variants in the presence of Doxycycline. Expression of the indicated PLCγ1 variants in HUVEC cells was analysed in the absence or presence of Doxycycline (bottom).

**Fig. 3:**
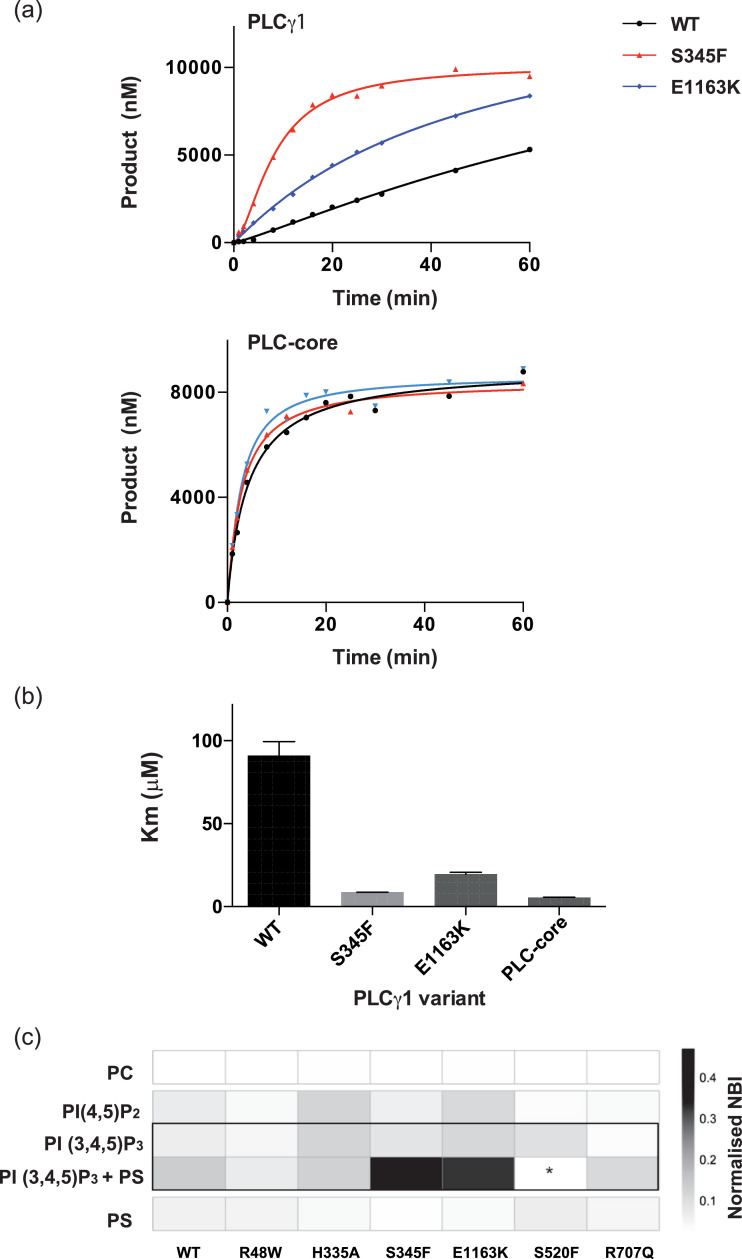
Properties of PLCγ1 variants *in vitro.* (a) Enzyme progress curves for PLCγ1 variants. PLC activity is indicated for WT (black), S345F (red) and E1163K (blue) variants of PLCγ1 in the context of full-length (top panel) and PLC−core (bottom panel). The substrate (PI) was presented as detergent/lipid mixed micelles. (b) Bar chart representing the Km values of the indicated variants determined using PLCglow as substrate and classic Michaelis-Menten kinetics. Kinetics were calculated using several concentrations of enzyme and the Km values calculated as the mean and SEM. (c) A heatmap representation of the binding of PLCγ1 variants to a range of phosphatidylinositol phosphate containing membranes. PI(4,5)P_2_ and PI(3,4,5)P_3_ were included at 10 mol% in liposomes formed from PC as the carrier lipid. DOPS was added at 10 mol% to test for cooperative effects. As controls, liposomes were formed from PC alone or from PC containing 10 mol% PS. The observed binding intensities were normalized, the mean value of at least 3 replicates is shown. Values with low reproducibility were excluded (*).

Our functional assessments, focused on the highlighted regions, included disease-linked mutations and additional deletions. Selected variants were analysed in a standard cellular assay and *in vitro* (Figs. 2a and 3). Furthermore, we extended our assessment of the hotspot mutations discovered in angiosarcoma to a physiologically relevant endothelial cell line where activation of PLCγ1 occurs in the context of RTK signalling (Fig. 2b and c).

The first group of mutations analysed in a cellular IP1 assay (Supplementary Figure S4) included mutations within the L1 and L3 loops of the TIM-barrel, namely, PLCγ1 S345F, Q1016L, Δ342–345, Δ1016–1019 and PLCγ2 D993G (Fig. 2a). Point mutations correspond to disease-linked variants while the deletions encompass regions with the highest mutation frequency. All mutations resulted an increase of PLC activity with the PLCγ1 S345F having the most prominent effect. Mutations in the C2 domain included PLCγ1 E1163K, D1165H, Δ1161–1166 and PLCγ2 M1141K. A further deletion in PLCγ1, Δ1164–1170, not corresponding to a precise disease-linked deletion, was also included. These mutations in the C2 domain all show a large increase in PLC activity. Disease-linked substitutions (PLCγ1 E730K and PLCγ2 A707P and S707Y) as well as a further PLCγ1 deletion (Δ730–733) in the cSH2 domain increased PLC activity although to a different degree. Similarly, disease-linked substitutions and additional deletions in the spPH domain (PLCγ1 S520F, H508R, Y509H, Δ865–869, Δ868–871 and PLCγ2 L848P) resulted in higher PLC activity.

In addition to the deletions described above (Fig. 2a), we also introduced larger deletions (Supplementary Figure S5). In contrast to the majority of mutations analysed in Fig. 2a that are further stimulated in the presence of EGF, the deletion of the regulatory region (ΔγSA or PLC-core) and the deletion of the segment with the critical Y783 (Δ776–790), as expected, lost their responsiveness to stimulation (Supplementary Figure S4). Furthermore, the fact that some other deletions (Δ1161–1166 and Δ1164–1170 in the C2 domain) also resulted in the loss of EGF stimulation suggests that the larger regions, unlike single specific residues within the region (E1163K), could incorporate complex roles affecting multiple aspects of the activation process.

Overall, our comprehensive analyses in a standard cell assay have revealed that the majority of tested mutations in the selected parts of PLCγ enzymes, irrespective of the disease and frequency of observations, resulted in an increase in enzyme activity. This functional analysis is consistent with previous findings reported for some of the mutations [6,8,15] and demonstrates the importance of the structural features with clustered mutations in controlling the activation state of the enzyme. The functional analyses also support the conclusion that the interacting surfaces revealed by structural studies have a role in autoinhibition.

We performed further, separate studies for mutations found in angiosarcoma, R707Q and S345F, particularly relevant for the RTK signalling. Interestingly, the R707 hotspot residue is not fully exposed on the cSH2 surface and the R707Q substitution could affect the stability of the cSH2 domain. Indeed, the measurements of thermal stability performed for the isolated WT cSH2 domain and the cSH2 domain with the R707Q substitution show a substantial decrease in stability in the latter (Fig. 2b, top). Measurements of basal PLC activity in a standard cellular assay show a relatively mild effect of R707Q compared to S345F substitution (Fig. 2b, bottom). Furthermore, when analysed in a cellular context relevant for angiosarcoma, in endothelial HUVEC cells, both mutations affect cellular morphology resulting in more elongated, spindling cells (Fig. 2c); this phenotype is similar to that seen after infection of HUVEC cultures with the Kaposi sarcoma virus (*i.e.* similar to sarcoma) [28]. Notably, the effect of R707Q is less pronounced and the impact of mutations correlates with the relative impact on the PLC activity assessed in a standard assay using a heterologous cell system (Fig. 2b).

### 3.3 Properties of activated PLCγ1 variants *in vitro*

Further characterization of disease-linked variants was performed *in vitro* (Fig. 3). We focused on substitutions in the PLC-core within the TIM-barrel surfaces close to the active site opening and surfaces on the C2 domain formed by the loop regions. As shown in Fig. 1e, in an inactive form they are occluded by the regulatory domain while in the active enzyme, these surfaces positioned within the same plane, are implicated in interactions with a cellular membrane. To show directly their importance in autoinhibition, we introduced S345F and E1163K substitutions in the context of the full-length PLCγ1 and in the context of the PLC-core where the regulatory specific array has been removed. Our data show an increase in PLC activity of the full-length PLCγ1 S345F and E1163K variants but not of the PLC-core with the same substitutions (Fig. 3a) and therefore demonstrate an impact on autoinhibition. Furthermore, the analysis of kinetic parameters shows that the full-length PLCγ1 S345F and E1163K variants have lower Km values (8.5 and 20.4 μM respectively) compared to the wild type (97 μM), that are similar to that of the PLC-core (5.6 μM) (Fig. 3b). These findings are also consistent with the effect of S345F and E1163K substitutions on autoinhibition and, subsequently, a greater accessibility of the active site.

As shown in Fig. 3a and b, the PLC assays based on detergent/lipid mixed micelles or a water soluble derivative of the substrate can measure PLC activity of the active site separately from the complexities of the substrate presentation within a cellular membrane. The activated forms of PLCγ1 could in addition to a greater accessibility of the active site also have different properties related to membrane binding. To test this possibility, we selected PLCγ1 variants where the PLC activity is affected to a different degree and assessed the interaction of purified proteins with a lipid microarray (LiMA) covering a range of lipid compositions [22]. As illustrated for a subset of conditions (Fig. 3c), proteins with the highest PLC activity show enhanced binding to a specific lipid combination, namely, PtdIns(3,4,5)P_3_ together with phosphatidylserine (PS). By adapting LiMA to score for the PLC activity (with low sensitivity), we demonstrated that S345F and E1163K variants retained their high activity in this setup (Supplementary Figure S6). Importantly, our finding that strongly activated variants also interact very strongly with membranes of this particular composition, are likely to reflect a conformational change in PLCγ1 that exposes surfaces with different lipid-binding properties rather than to simply increase hydrophobicity of the membrane-interacting area. Our data showing that activating mutations affecting different domains in PLCγ1 have the same membrane binding properties (S345F in the TIM-barrel and E1163K in the C2 domain) further suggest that these properties reflect a common change in conformation of an active enzyme rather than specific interactions involving particular substitutions.

Together, our data presented in Fig. 3 suggest that activation of PLCγ1 by the two key mutations involves a release of auto-inhibition resulting in the increased exposure of the active site and also in changes in membrane-binding properties. They together contribute to an increase in PLCγ1 activity observed in cells.

### 3.4 Further characterization of interactions between an intact PLCγ1 and FGFR1

**Fig. 4:**
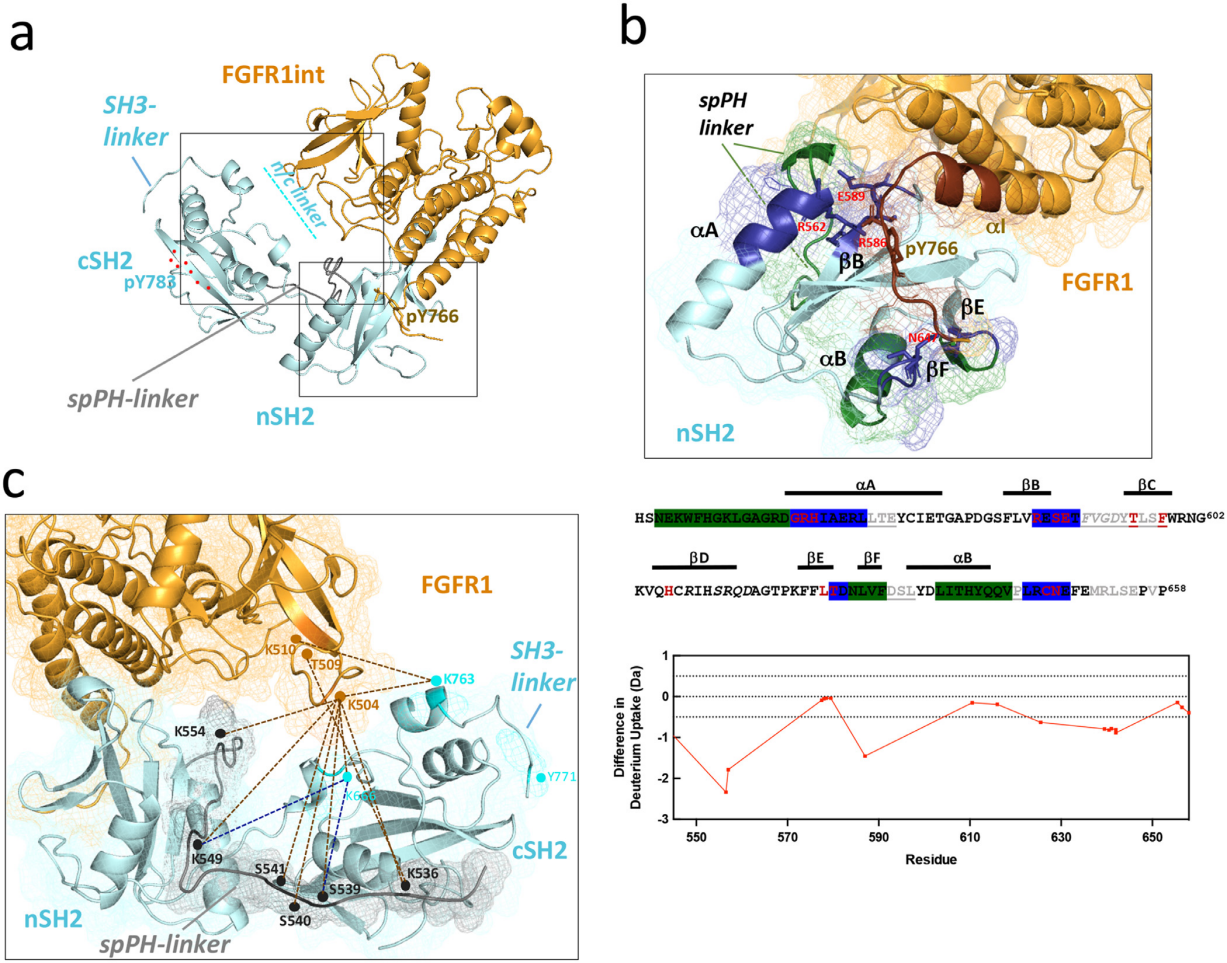
Interaction between an intact PLCγ1 and FGFR1int, analysed using HDX-MS and XL-MS. (a) Structure of the PLCγ1 SH2 tandem/FGFRint complex (PDB 3GQI) including a position of the spPH linker (connecting spPH and nSH2; grey) based on XL-MS data. Segment marked with a red dotted line is a part of the SH3 linker (connecting cSH2 and SH3) containing pY783, when bound to the cSH2 domain in an active form of PLCγ1 (PDB 4EY0). The two areas marked by rectangles are shown as close up in parts b and c. (b) A close up of the strong, canonical binding site (top). The HDX-MS data are presented so that the peptides that become protected in the nSH2 domain are coloured dark blue (direct binding) or green (outside direct binding) and those in FGFR1int are coloured brown. In the sequence of the nSH2 domain (bottom), the regions that show significant change are highlighted using the same colour coding and interacting residues identified in the 3GQI structure indicated. Underlined sequences in grey correspond to peptides not covered by the HDX-MS analysis. The difference in deuterium uptake for the nSH2 is presented in the graph below. (c) A close up of the complex in the vicinity of the N-lobe of FGFR1 kinase. Residues and distances identified by XL-MS for the PLCγ1/FGFRint complex are indicated in the 3GQI structure. All major cross-links for FGFR1int interactions with PLCγ1 are as brown dotted lines. The XL-MS data also show proximity of a part of spPH-nSH2 linker with both FGFRint (brown dotted lines) and nSH2-cSH2 (n/c) linker (blue dotted lines) in PLCγ1 and have provided the information to model this linker (grey). (For interpretation of the references to color in this figure legend, the reader is referred to the web version of this article.)

In addition to the insights obtained from cryo-EM (Fig. 1e), we examined interactions between the FGFR1int and an intact PLCγ1 in solution and applied XL-MS and HDX-MS (Fig. 4, Supplementary Figures S7 and S8). The two methods are complementary and while HDX-MS defines the changes in exposure of a particular protein segment due to either protein-protein interactions or to a conformational change, the XL-MS provides constraints for the distances between the cross-linked amino acid residues [29,30].

Our HDX-MS data confirm that the main binding pocket is represented by canonical and secondary interaction sites in the nSH2 domain that strongly bind the FGFR within the C-terminal tail containing pY766 and surrounding residues (Fig. 4b and Supplementary Figure S8). Based on XL-MS, a number of crosslinks (in particular between the β2-β3 linker/β3 in FGFR1 and nSH2-cSH2linker/cSH2 in PLCγ1) are also consistent with the relative position of the FGFR1 kinase N-lobe and the PLCγ1 SH2 tandem in the crystal structure (PDB 3GQI). Furthermore, our data also show the proximity of the spPH-nSH2 linker to both, FGFR1 β2-β3 linker/β3 and the cSH2-nSH2 linker. These relative arrangements and their proximity suggest that, in addition to the strong binding site, the FGFR1 kinase and PLCγ1 interactions could also include weaker and transient bindings sites in these flexible and solvent exposed regions (Fig. 4c).

HDX-MS data also show changes in exposure (protection) of different parts of nSH2 that are outside the key interaction area with FGFR1 (Fig. 4b and Supplementary Figure S8). In particular, changes in parts of the αB and in the spPH-nSH2 linker adjacent to αA (dark green) could represent a conformational change subtly influencing interactions between the regulatory domains.

## 4 Discussion

Our structural studies and further supporting evidence define the architecture of an intact PLCγ1 in an auto-inhibited form and of the complex with a known upstream regulator, the kinase domain and proximal C-terminal tail from FGFR1. Key to the understanding of regulation and deregulation of the PLCγ family are two interfaces, namely, the interface between the PLC-core and regulatory region in PLCγ and the interface between the FGFR1 and the PLCγ regulatory region. Previous studies outlined some of the interactions that could be taking place within these interfaces [18], [19], [20] but were insufficient to provide a complete picture required to define molecular mechanisms.

We obtained cryo-EM data at an intermediate resolution and to exclude any unambiguity of the model we provided further confirmation by applying HDX-MS, XL-MS and mutational analyses. Further insights at atomic level would require a high-resolution structure. Nevertheless, we here show that the PLC-core in an autoinhibited PLCγ is in the proximity of several regulatory domains that prevent access to the membrane and its lipid substrate (Fig. 1e and f). Of particular importance are surfaces on the catalytic TIM-barrel and C2 domain of the PLC-core, positioned close to the spPH and cSH2 regulatory domains, respectively. Mutations within either of the two contact points can overcome the enzyme autoinhibition (Fig. 2 and 3) implying that both contacts are required to maintain PLCγ in an inhibited form. The extensive occlusion of the PLC-core by the regulatory domains is unique to the PLCγ family and illustrates the diversity of regulatory mechanisms among PLC enzymes [31].

From the structure of the autoinhibited form, it is clear that for activation of PLCγ enzymes, conformational changes that expose regions implicated in membrane interactions need to take place. Previous studies have suggested that for the physiological activation mediated by phosphorylation of Y783 in the cSH2-SH3 linker, the intra-molecular interaction between pY783 and the cSH2 domain results in the release of autoinhibition and, in turn, the exposure of the membrane interacting regions [19]. This is consistent with the structure of the inactive PLCγ1 where the phosphopeptide binding site of the cSH2 domain is oriented towards the C2 domain at the autoinhibitory interface (Fig. 1e and Supplementary Figure S2). However, it is not clear how interactions with the kinase result in concomitant phosphorylation of Y783 and changes that bring together pY783 and its binding site on the cSH2 domain. This is likely to be mediated by the allosteric networks formed by the elements from the regulatory region and could involve conformational changes in the nSH2 domain resulting from strong binding, together with transient interactions (Fig. 4).

**Fig. 5:**
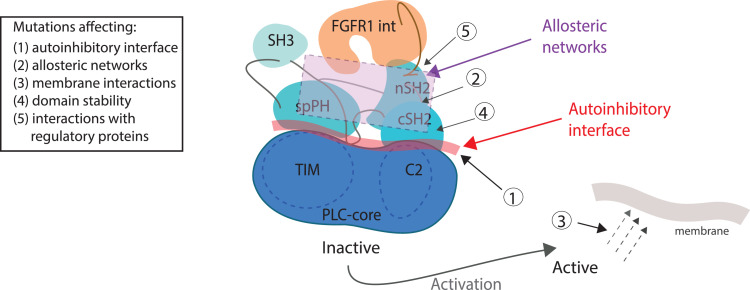
Mechanistic implications of the PLCγ architecture. The inactive form of PLCγ is maintained by interactions at the extensive autoinhibitory interface. In order to release the autoinhibition, physiological stimulation, impacting on the distant parts of the regulatory region, is propagated *via* allosteric networks to the autoinhibitory interface. In the case of phosphorylation, changes in such networks are needed to bring about the intramolecular interaction between the phosphorylated segment and the cSH2 domain at the autoinhibitory interface. Allosteric networks could also have a role to stabilize the active form. Based on this framework, it is possible to suggest different mechanistic classes of mutations listed here (box, left). The majority of mutations are part of or in the vicinity of the autoinhibitory interface and directly affect the autoinhibition. Several examples also support other mechanisms included in the list (box, left). The features related to different mechanistic classes are indicated by arrows (diagram, right).

Importantly, the overall organisation of the PLCγ1/FGFRint complex, involving an extensive autoinhibitory interface in PLCγ1 linked to an upstream signalling protein via allosteric networks, provide a valuable framework for mechanistic interpretation of various mutations found in diverse pathologies (Fig. 5). Our studies of the distribution and functional impact of mutations show that the number of very frequent mutations and less frequent mutations clustered in the same regions, increase PLC activity by directly affecting autoinhibitory interactions (Figs. 2 and 3 and Supplementary Figure S3). The mutated residues that belong to this mechanistic class include PLCγ1 S345, E1163, D1165, and S520 reported in T-cell lymphoma, with the PLCγ1 S345F mutation also found in angiosarcoma. Several frequently mutated residues in Ibrutinib-resistant CLL, including PLCγ2 S707, L845, D993 and M1141, are likely to impact on PLC activity *via* the same mechanism. Interestingly, some of the somatic mutations in the resistant CLL are identical to genetic lesions found in APLAID and include mutations of PLCγ2 S707 and M1141 residues [6,15,32,33]. However, not all mutations reside within, or in the vicinity, of the intramolecular inhibitory interactions, suggesting other mechanistic classes. For example, we have recently shown that one frequent mutation in the resistant CLL, PLCγ2 R665W, is likely to stabilize an active form by affecting allosteric networks to facilitate binding of the phosphorylated peptide to the cSH2 domain [34]. In this study we provide another example of a different mechanism; a frequent PLCγ1 R707Q substitution found in angiosarcoma decreases stability of the cSH2 domain and in this indirect way could affect the autoinhibition (Fig. 2b). An additional mechanistic class could be related to modulation of membrane interactions of an active PLCγ. The PLCγ1 R48W mutation frequently found in T-cell lymphoma affects the nPH domain, implicated in inositol-lipid binding, and provides a possible candidate for this mechanism.

In conclusion, our new insights reveal features of PLCγ enzymes that are important for determining their activation status. Targeting such features, as an alternative to targeting the PLC active site that has so far not been achieved for any PLC, could provide new routes for clinical interventions related to various pathologies driven by PLCγ deregulation.

## Funding sources

MK acknowledges support from CR UK (A16567) and MRC (P028160). The funders, CR UK, MRC and AstraZeneca, had no role in the writing of the manuscript or the decision to submit it for publication. The corresponding author had full access to all the data in the study and had final responsibility to for the decision to submit for publication.

## Declaration of interests

AC-G and KB report financial research support from AbbVie Deutschland GmbH & Co. KG, outside the submitted work. CP, CS, TMO and YL report that they are employees or former employees of AstraZeneca without a conflict of interest related to the work reported in this manuscript. All other authors have nothing to disclose.

## Appendix Supplementary materials

Image, application 1

Image, application 2
