# Supplementary material for: Structural insights and activating mutations in diverse pathologies define mechanisms of deregulation for phospholipase C gamma enzymes
Source: eBioMedicine. 2020 Jan 6;51:102607. doi: 10.1016/j.ebiom.2019.102607 (PMC7000336; doi:10.1016/j.ebiom.2019.102607)
Supplement: Supplementary file 1 [file mmc1.pdf]

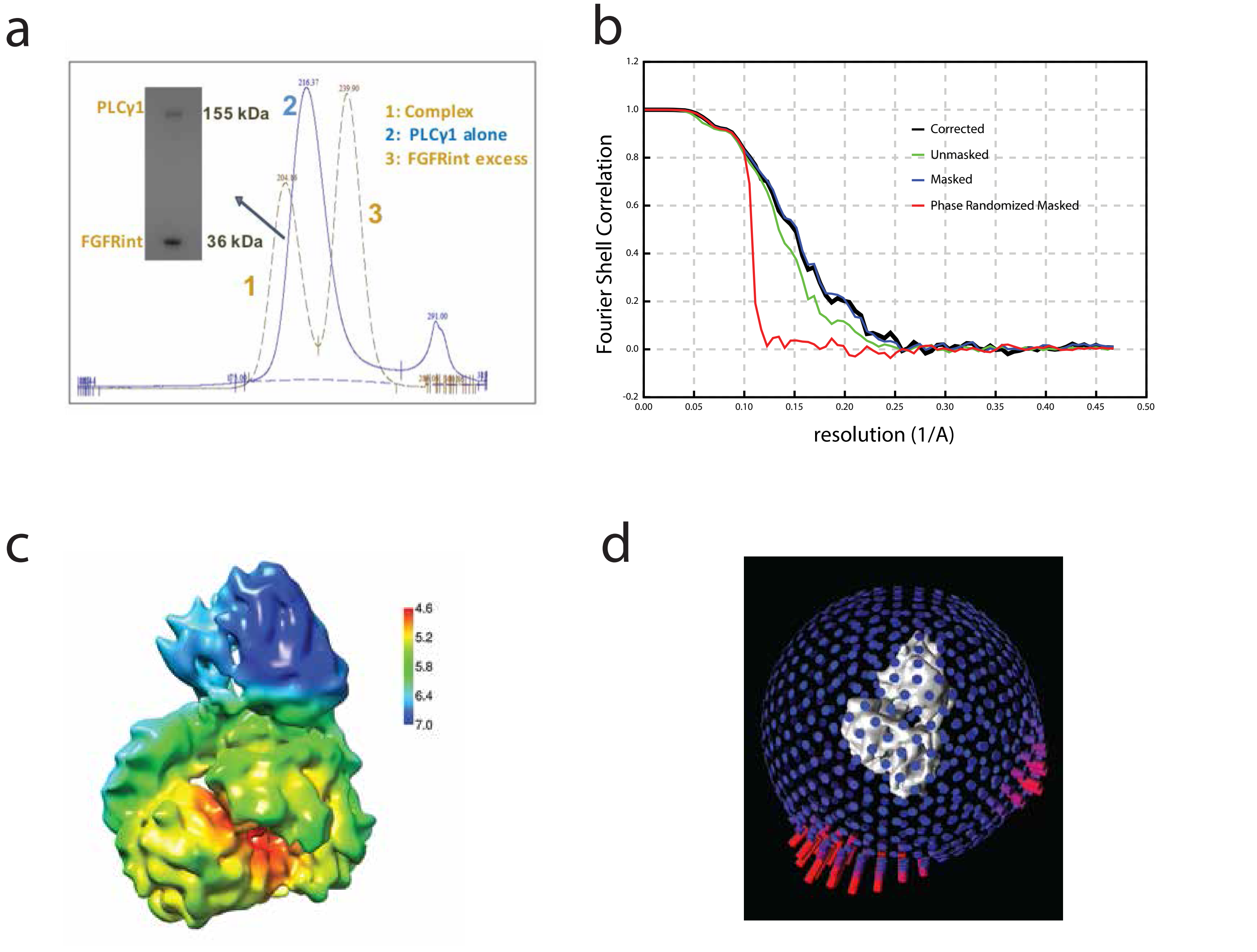
a
PLCγ1
155 kDa
FGFRint
36 kDa
2
1: Complex
2: PLCγ1 alone
3: FGFRint excess
3
1
b
Fourier Shell Correlation
resolution (1/A)
Corrected
Unmasked
Masked
Phase Randomized Masked
c
4.6
5.2
5.8
6.4
7.0
d

Figure S1

a

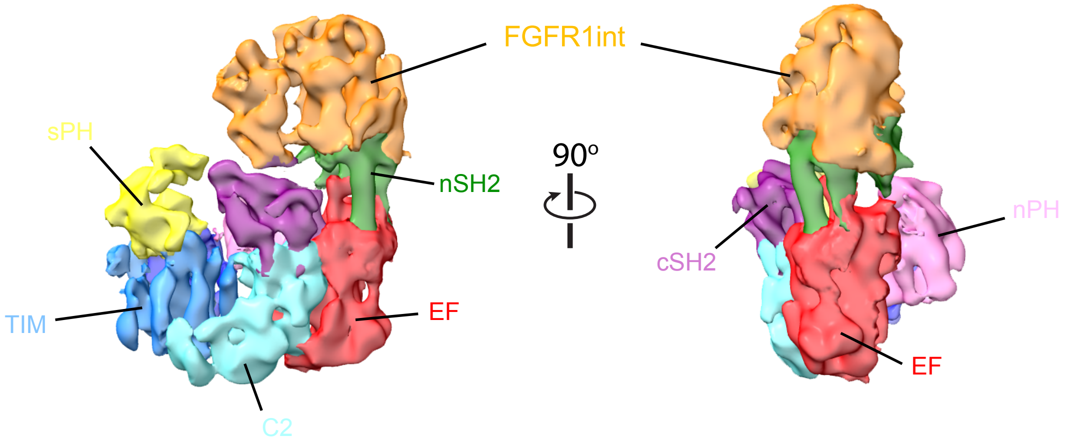

b

Domain	σ level	CC value
nPH	0.025	0.8066
EF	0.025	0.7768
TIM-X	0.03	0.8329
sPH	0.02	0.8230
nSH2	0.025	0.8647
cSH2	0.02	0.8173
TIM-Y	0.03	0.8476
C2	0.025	0.8472
FGFR1int	0.02	0.8092

c

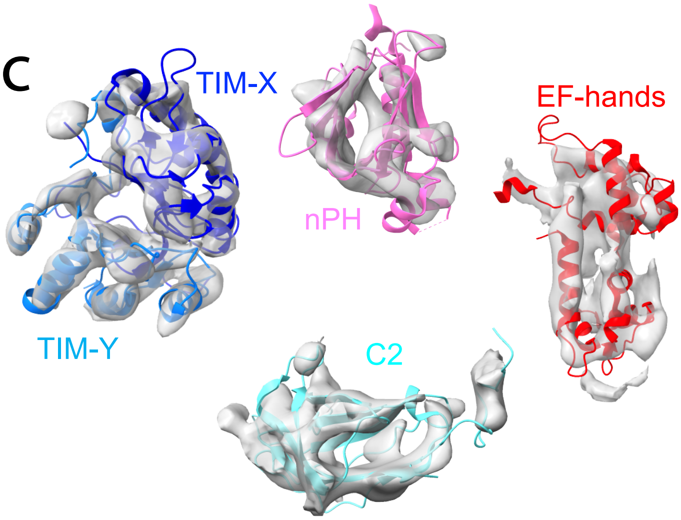

d

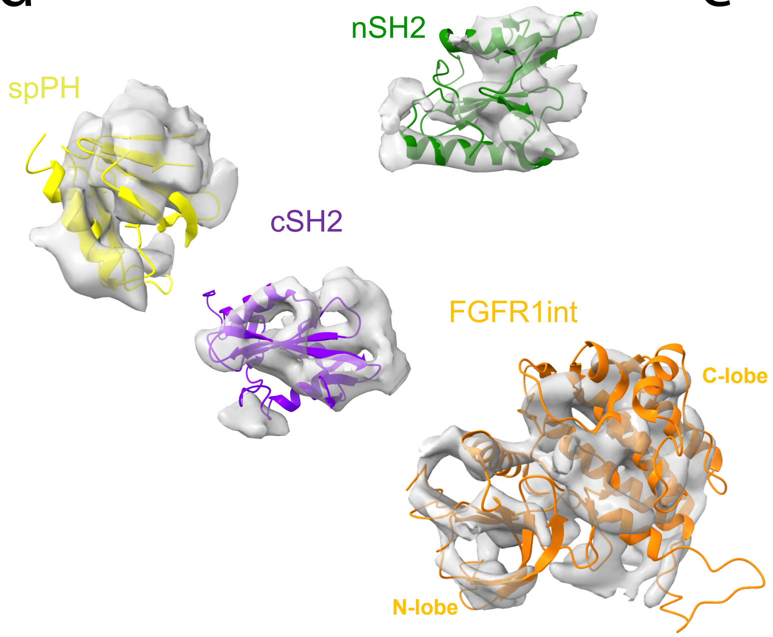

e

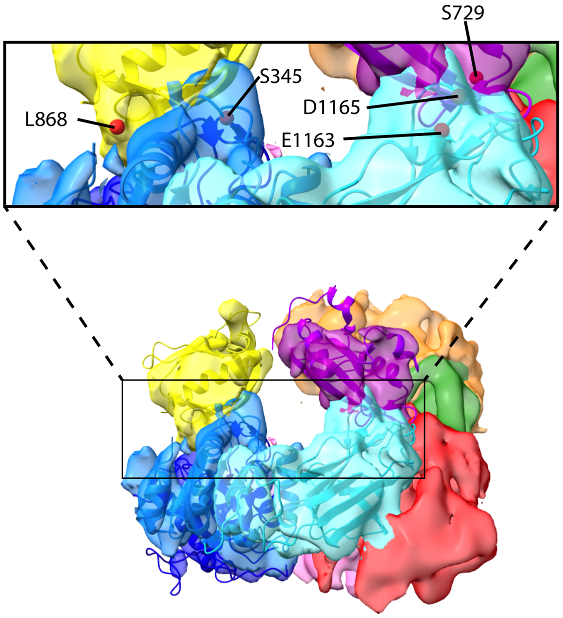

Figure S2

a

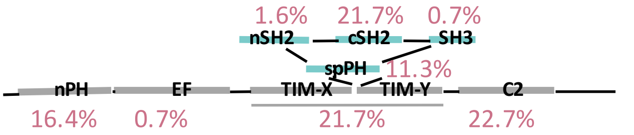

Total number of observations = 432

```
                           nPH
PLCG1 MAGAASPCANGCGPGAPSDAEVLHLCRSLEVGTVMTLFYSKKSQRPERKTFQVKLETRQI 60
PLCG2 -------MSTTVNVDSLAEYEKSQIKRALELGTVMTVFSFR-KSTPERRTVQVIMETRQV 52

PLCG1 TWSRGADKIEGAIDIREIKEIRPGKTSRDFDRYQEDPAFRPDQSHCFVILYGMEFRLKTL 120
PLCG2 AWSKTADKIEGFLDIMEIKEIRPGKNSKDFERA---KAVRQKEDCCFTILYGTQFVLSTL 109
                                          EF
PLCG1 SLQATSEDEVNMWIKGLTWLMEDTLQAPTPLQIERWLRKQFYSVDRNREDRISAKDLKNM 180
PLCG2 SLAADSKEDAVNWLSGLKILHQEAMNASTPTIIESWLRKQIYSVDQTRRNSISLRELKTI 169

PLCG1 LSQVNYRVPNMRFLRERLTDLEQRSGDITYGQFAQLYRSLMYSAQKTMDLPFLEAS---T 237
PLCG2 LPLINFKVSSAKFLKDKFVEIGAHKDELSFEQFHLFYKKLMFEQQKSILDEFKKDSSVFI 229

PLCG1 LRAGERPELCRVSLPEFQQFLLDYQGELWAVDRLQVQEFMLSFLRDPLREIEEPYFFLDE 297
PLCG2 LGNTDRPDASAVYLHDFQRFLIHEQQEHWAQDLNKVRERMTKFIDDTMRETAEPFLFVDE 289
                                        TIM-X
PLCG1 FVTFLFSKENSVWNSQLDAVCPDTMNNPLSHYWISSSHNTYLTGDQFSSESSLEAYARCL 357
PLCG2 FLTYLFSRENSIWDEKYDAVDMQDMNNPLSHYWISSSHNTYLTGDQLRSESSPEAYIRCL 349

PLCG1 RMGCRCIELDCWDGPDGMPVIYHGHTLTTKIKFSDVLHTIKEHAFVASEYPVILSIEDHC 417
PLCG2 RMGCRCIELDCWDGPDGKPVIYHGWTRTTKIKFDDVVQAIKDHAFVTSSFPVILSIEEHC 409
                                                            ------
PLCG1 SIAQQRNMAQYFKKVLGDTLLTKPVEISADGLPSPNQLKRKILIKHKKLAEGSAYEEVPT 477
PLCG2 SVEQQRHMAKAFKEVFGDLLLTKPTEASADQLPSPSQLREKIIIKHKKLGPRGDVDV--- 466
      -------     spPH-p1
PLCG1 SMMYSENDISNSIKNGILYLEDPVNHEWYPHYFVLTSSKIYYSEETSSDQGNEDEEEPKE 537
PLCG2 ---NMEDKKDEHKQQGELYMWDSIDQKWTRHYCAIADAKLSFSDDIEQTM----EEEVPQ 519
                  nSH2
PLCG1 VSSSTELHSNEKWFHGKLGAGRDGRHIAERLLTEYCIETGAPDGSFLVRESETFVGDYTL 597
PLCG2 DIPPTELHFGEKWFHKKVEK----RTSAEKLLQEYCMETGGKDGTFLVRESETFPNDYTL 575

PLCG1 SFWRNGKVQHCRIHSRQDAGTPKFFLTDNLVFDSLYDLITHYQQVPLRCNEFEMRLSEPV 657
PLCG2 SFWRSGRVQHCRIRSTMEGGTLKYYLTDNLTFSSIYALIQHYRETHLRCAEFELRLTDPV 635
                cSH2
PLCG1 PQTNAHESKEWYHASLTRAQAEHMLMRVPRDGAFLVRKRNEPNSYAISFRAEGKIKHCRV 717
PLCG2 PNPNPHESKPWYYDSLSRGEAEDMLMRIPRDGAFLIRKREGSDSYAITFRARGKVKHCRI 695
                 --
PLCG1 QQEGQTVMLGNSE-FDSLVDLISYYEKHPLYRKMKLRYPINEEALEKIGTAEPDYGALYE 776
PLCG2 NRDGRHFVLGTSAYFESLVELVSYYEKHSLYRKMRLRYPVTPELLERYNME-RDINSLYD 754
                  SH3
PLCG1 GRNPGFYVEANP--MPTFKCAVKALFDYKAQREDELTFIKSAIIQNVEKQEGGWWRGDYG 834
PLCG2 VS--RMYVDPSEINPSMPQRTVKALYDYKAKRSDELSFCRGALIHNVSKEPGGWWKGDYG 812
                                         spPH-p2
PLCG1 GKKQLWFPSNYVEEMVNPVALEPEREHLDENSPLGDLLRGVLDVPACQIAIRPEGKNNRL 894
PLCG2 TRIQQYFPSNYVEDISTAD-FEELEKQIIEDNPLGSLCRGILDLNTYNVVKAPQGKNQKS 871
                                                           TIM-Y
PLCG1 FVFSISMASVAHWSLDVAADSQEELQDWVKKIREVAQTADARLTEGKIMERRKKIALELS 954
PLCG2 FVFILEPKQQGDPPVEFATDRVEELFEWFQSIREITWKIDTKENNMKYWEKNQSIAIELS 931

PLCG1 ELVVYCRPVPFDEEKIGTERACYRDMSSFPETKAEKYVNKAKGKKFLQYNRLQLSRIYPK 1014
PLCG2 DLVVYCKPTSKTKDN--LENPDFREIRSFVETKADSIIR-QKPVDLLKYNQKGLTRVYPK 988

PLCG1 GQRLDSSNYDPLPMWICGSQLVALNFQTPDKPMQMNQALFMTGRHCGYVLQPSTMRDEAF 1074
PLCG2 GQRVDSSNYDPFRLWLCGSQMVALNFQTADKYMQMNHALFSLNGRTGYVLQPESMRTEKY 1048
                C2
PLCG1 DPFDKSSLRGLEPCAISIEVLGARHLPKNGRGIVCPFVEIEVAGAEYDSTKQKTEFVVDN 1134
PLCG2 DPMPPESQRKIL-MTLTVKVLGARHLPKLGRSIACPFVEVEICGAEYDNNKFKTTVVNDN 1107
                                   ------  -----
PLCG1 GLNPVWPAK--PFHFQISNPEFAFLRFVVYEEDMFSDQNFLAQATFPVKGLKTGYRAVPL 1192
PLCG2 GLSPIWAPTQEKVTFEIYDPNLAFLRFVVYEEDMFSDPNFLAHATYPIKAVKSGFRSVPL 1167

PLCG1 KNNYSEDLELASLLIKIDIFPAKENGDLSPFSGTSLRERGSDASGQLFHGRAREGSFESR 1252
PLCG2 KNGYSEDIELASLLVFCEMRPVLESEEELYSSCRQLRRRQEELNNQLFLYDTHQNLRNAN 1227

PLCG1 YQQPFEDFRISQEHLADHFDSRERRAPRRTRVNGDNRL- 1290
PLCG2 RDALVKEFSVNENQLQL-YQEKCNKRLREKRVSNSKFYS 1265
```

Number of reported mutations for a given residue	
	1-3
	4-15
	16-30
	31-80

Rare variants

b

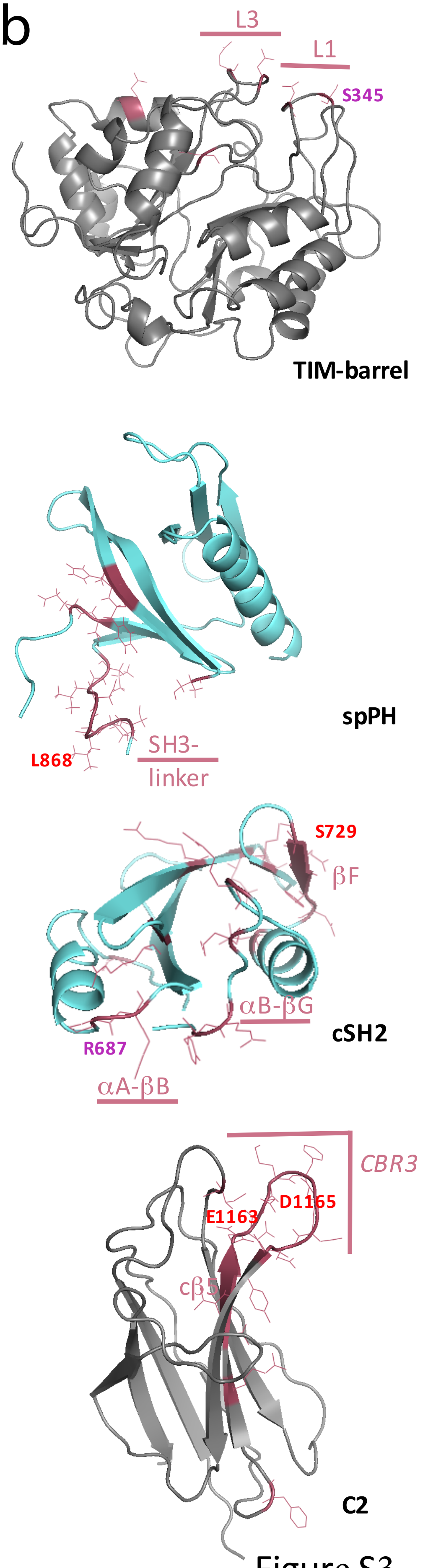

Figure S3

a

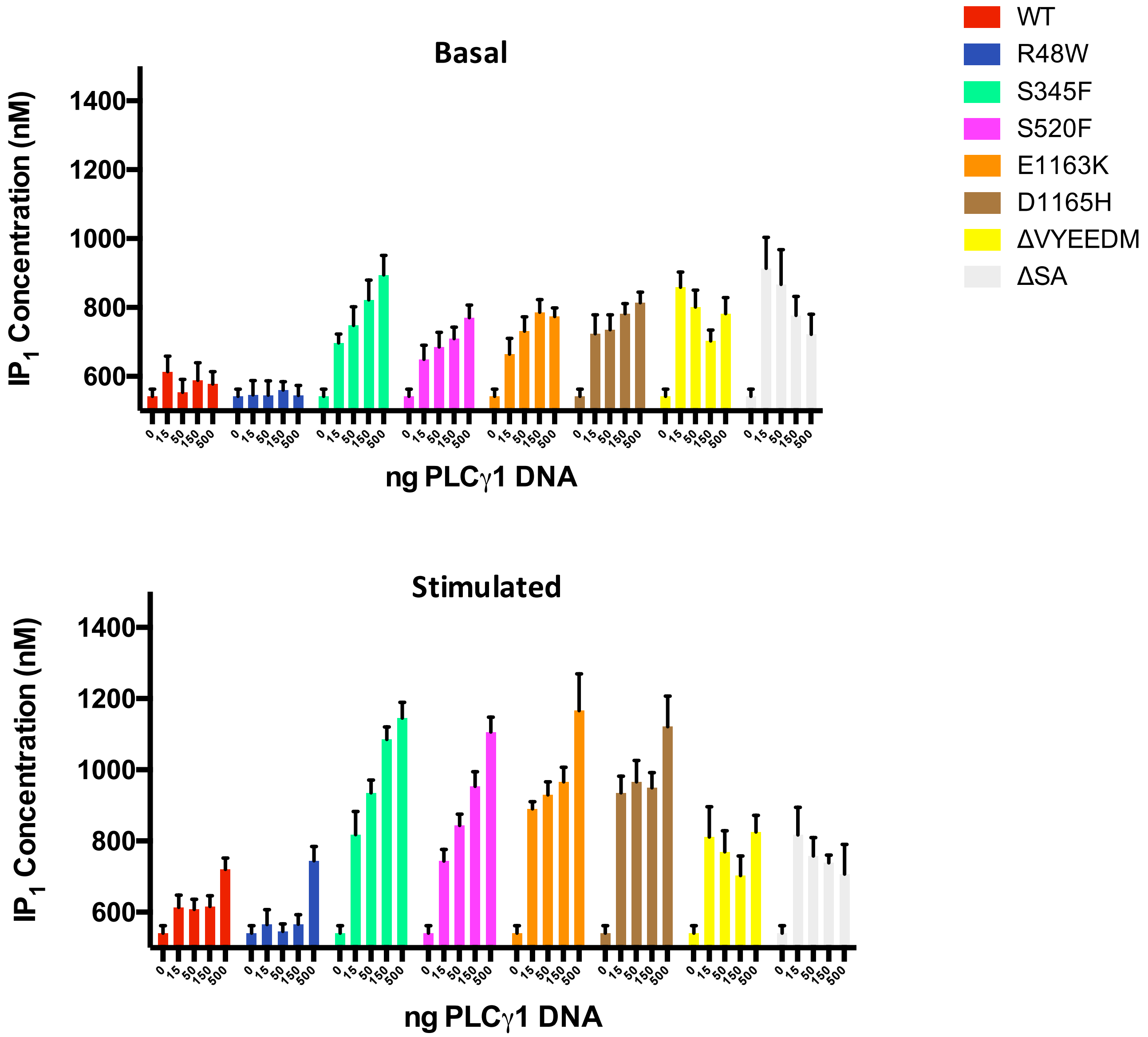

Basal
Stimulated
IP1 Concentration (nM)
1400
1200
1000
800
600
ng PLCγ1 DNA
0
15
50
150
500
WT
R48W
S345F
S520F
E1163K
D1165H
ΔVYEEDM
ΔSA

b

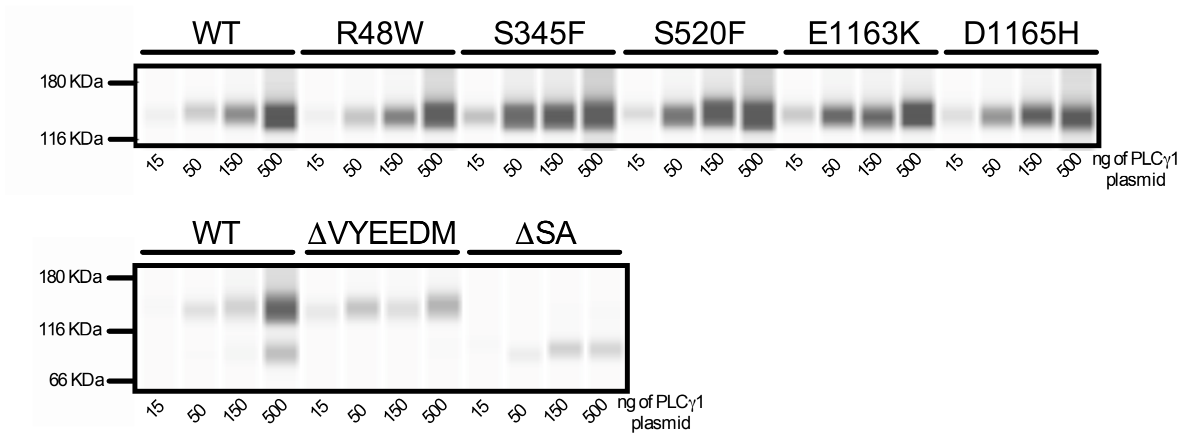

WT
R48W
S345F
S520F
E1163K
D1165H
ΔVYEEDM
ΔSA
180 KDa
116 KDa
66 KDa
15
50
150
500
ng of PLCγ1 plasmid

Figure S4

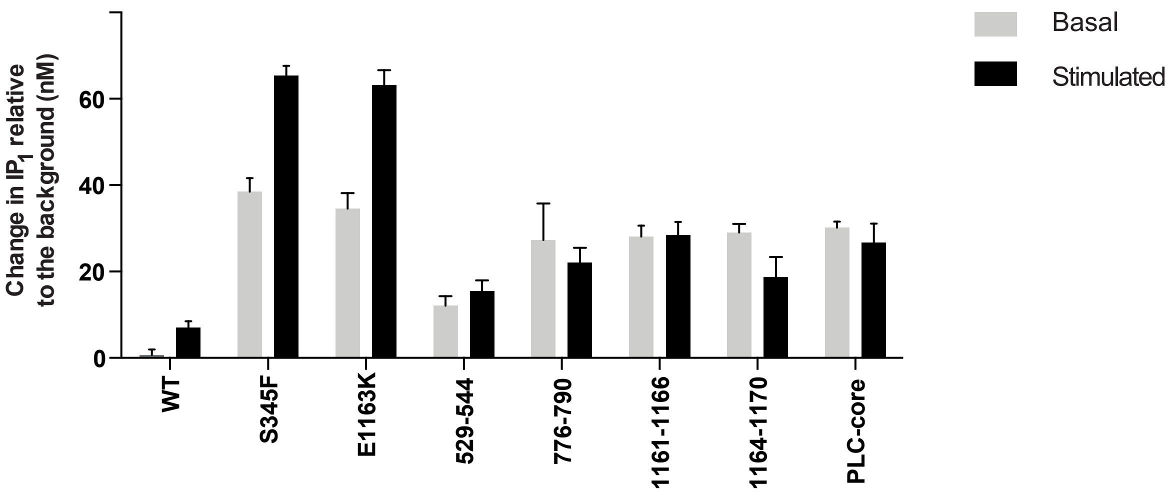
Basal
Stimulated
Change in IP_1 relative to the background (nM)
0
20
40
60
WT
S345F
E1163K
529-544
776-790
1161-1166
1164-1170
PLC-core

Figure S5

a

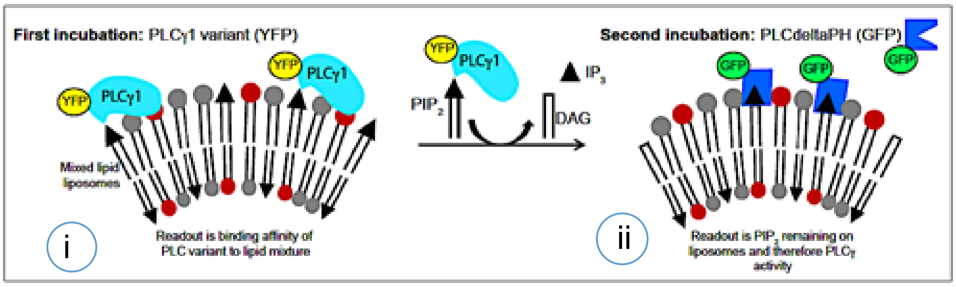

First incubation: PLCγ1 variant (YFP)
YFP
PLCγ1
Mixed lipid liposomes
i
Readout is binding affinity of PLC variant to lipid mixture
PIP2
IP3
DAG
Second incubation: PLCdeltaPH (GFP)
GFP
ii
Readout is PIP2 remaining on liposomes and therefore PLCγ activity

b

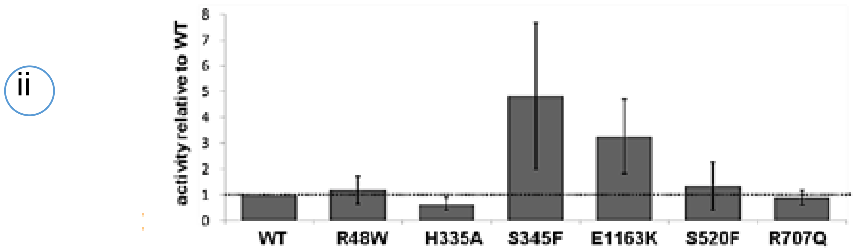

ii
activity relative to WT
0
1
2
3
4
5
6
7
8
WT
R48W
H335A
S345F
E1163K
S520F
R707Q

Figure S6

a

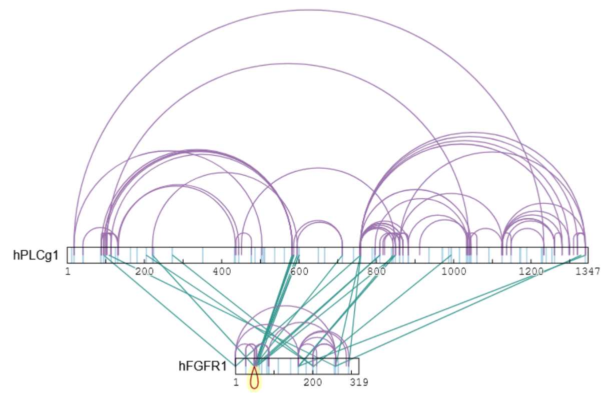

hPLCg1
1
200
400
600
800
1000
1200
1347
hFGFR1
1
200
319

b

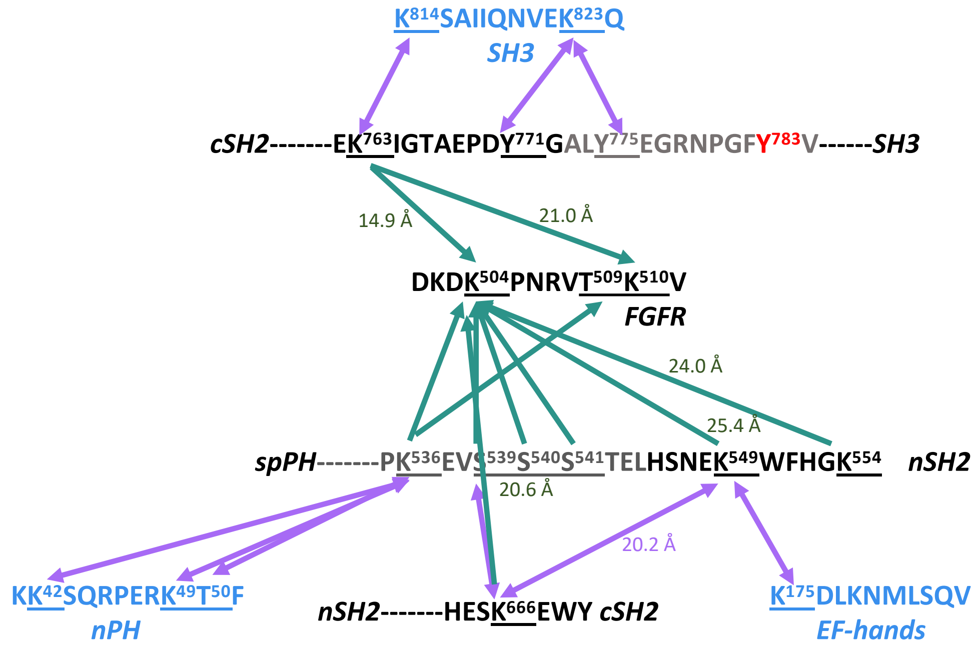

K814SAIIQNVEK823Q
SH3
cSH2-------EK763IGTAEPDY771GALY775EGRNPGFY783V------SH3
14.9 Å
21.0 Å
DKDK504PNRVT509K510V
FGFR
24.0 Å
25.4 Å
spPH-------PK536EVS539S540S541TELHSNEK549WFHGK554
nSH2
20.6 Å
20.2 Å
KK42SQRPERK49T50F
nPH
nSH2-------HESK666EWY cSH2
K175DLKNMLSQV
EF-hands

Figure S7

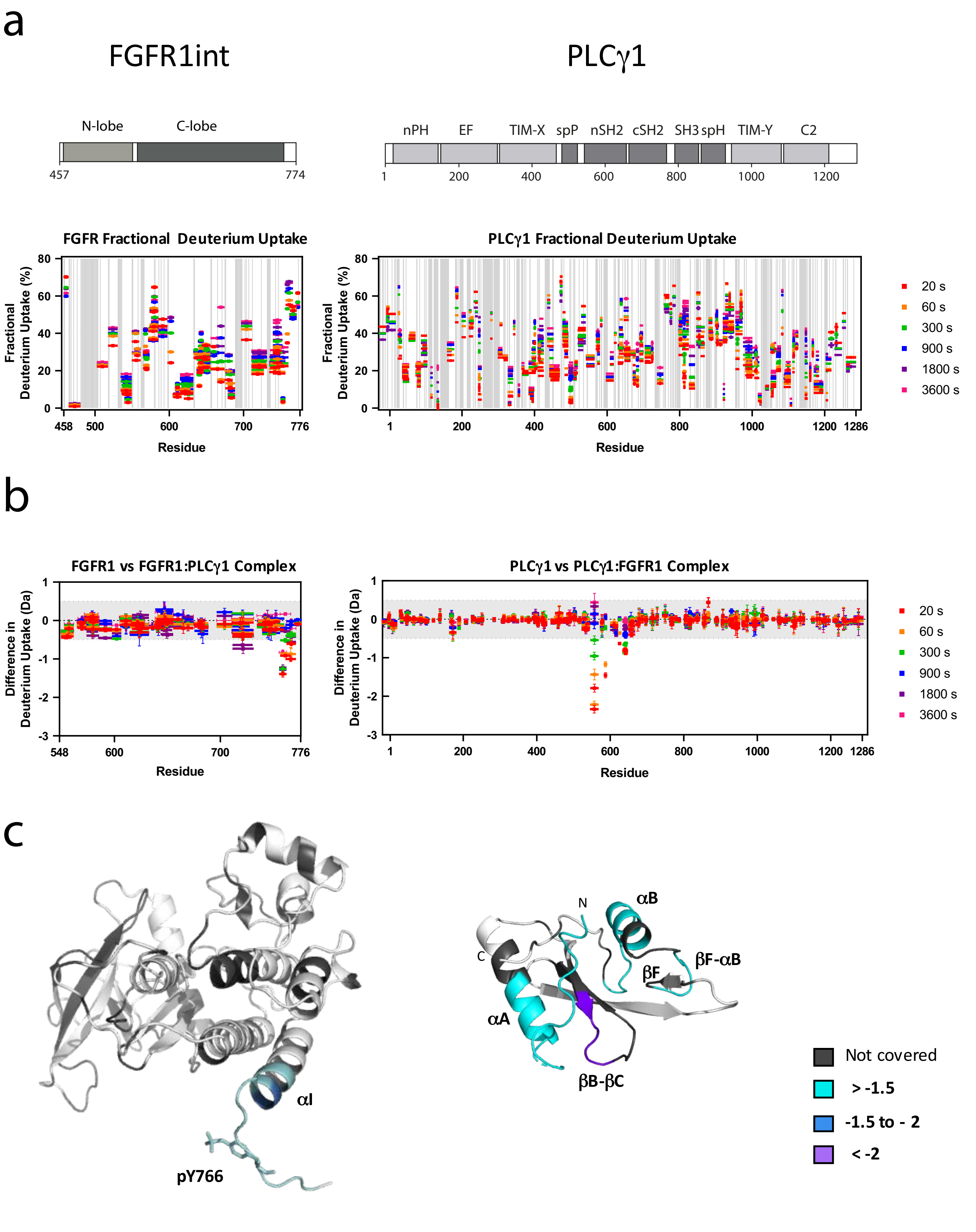
a
FGFR1int
PLCγ1
N-lobe
C-lobe
457
774
nPH
EF
TIM-X
spP
nSH2
cSH2
SH3
spH
TIM-Y
C2
FGFR Fractional Deuterium Uptake
PLCγ1 Fractional Deuterium Uptake
Fractional Deuterium Uptake (%)
Residue
20 s
60 s
300 s
900 s
1800 s
3600 s
b
FGFR1 vs FGFR1:PLCγ1 Complex
PLCγ1 vs PLCγ1:FGFR1 Complex
Difference in Deuterium Uptake (Da)
Residue
c
αI
pY766
N
C
αB
βF
βF-αB
αA
βB-βC
Not covered
> -1.5
-1.5 to - 2
< -2

Figure S8
