# Supplementary material for: Structural insights and activating mutations in diverse pathologies define mechanisms of deregulation for phospholipase C gamma enzymes
Source: eBioMedicine. 2020 Jan 6;51:102607. doi: 10.1016/j.ebiom.2019.102607 (PMC7000336; doi:10.1016/j.ebiom.2019.102607)
Supplement: Supplementary file 2 [file mmc2.pdf]

SUPPLEMENTARY FIGURE LEGENDS

Figure S1. Protein preparation and Cryo-EM processing.
(a) Size exclusion chromatogram representing the purification of the complex of PLCγ1/FGFRint along with a coomassie stained SDS-PAGE analysis of the purified complex.
(b) FSC plot.
(c) Representation of the estimated local resolution of the 3D cryoEM map.
(d) Angular distribution plot.

Figure S2. Cryo-EM map fitting.
(a) Two different views of the cryo-EM map with the density of individual domains differently coloured (prepared in ChimeraX).
(b) Table of cross-correlation coefficient (CC) values. This panel reports information about the fitting shown in (c) and (d), with the σ level of the map and the CC value for each domain. The CCs were calculated using CHIMERA by comparing each map segment against a map generated from the corresponding PDB crystal/NMR structure at 6 Å. The CC values within the same range were obtained with Phenix or when partial, multi-domain structures (PDB 4QJ3 or the PLCγ1 core homology model and PDB 3GQI) were used.
(c) Representation of individual domain fitting for the PLCγ1 core. Domain structures are taken from PDB 4QJ3. The colour-coding is the same as in (a).
(d) Representation of individual domain fitting for the PLCγ1 specific array and FGFR1int. Domain structures are taken from PDB 3GQI (for FGFR1int, nSH2 and cSH2) and PDB 2FJL (for split PH). The colour-coding is the same as in (a).
(e) Representation of the cryoEM map as in (a) but focussed on the autoinhibitory interface formed between the TIM/split PH and C2/cSH2 domains. The inset shows a close-up of this interface with the position of amino acid residues involved in PLCγ1 pathologies indicated.

Figure S3. Mutations in PLCγ enzymes discovered in diverse pathologies.
(a) Distribution of mutations across PLCγ1 domains presented as frequency (top diagram). A sequence alignment of PLCγ1 and PLCγ2 including the different domains highlighted in grey and cyan. The mutations (single amino acid substitutions and deletions) include those reported for PLCγ1 and PLCγ2 in genetic studies covering the main relevant pathologies: T-cell lymphomas, angiosarcoma, Btk-inhibitor resistance in CLL and various immune disorders. Total number of observations = 432.
Individual residues are highlighted in the PLCγ1 and PLCγ2 sequence and colour-coded based on their frequencies as shown in the legend below.
Resources used to perform the analysis included COSMIC database and original publications listed in Supplemental References.
(b) Individual residues, reported to be mutated, are also shown in structures of PLCγ1 TIM-barrel (modelled from PDB 4QJ3), spPH (PDB 2FJL), cSH2 (PDB 4FBN) and C2 (modelled from PDB 4QJ3) domains (represented as pink lines) and regions that show clustering of the mutated residues indicated and labelled. Frequently mutated residues are also labeled (frequencies 16-30 in red and 31-80 in purple) using PLCγ1 numbering.

Figure S4. Cellular assay for PLC activity in transfected cells.

(a) Measurement of the production of inositol phosphate in transfected COS-7 cells is illustrated for a subset of the PLCγ1 variants shown in main Figure 2. For each variant, plasmid DNA (0-500 ng) is titrated and the cells treated in the absence (basal) or presence of EGF (stimulated). The data represent experimental duplicates of biological triplicates and the values are presented as the mean with error bars showing the SEM.

(b) Protein expression of the indicated PLCγ1 variants using titration with an increasing amount of plasmid DNA was determined by WES (Protein Simple).

For the data shown in Figure 2a and Supplemental Figure S5, all variants were tested using titrations as shown here and the PLC activities corresponding to the same PLCγ1 expression levels included in direct comparison.

Figure S5. Comparison of PLC activity for selected PLCγ1 variants in the absence and presence of EGF stimulation.

PLC activity in transfected COS-7 cells using the IP_1 assay for the indicated variants of PLCγ1. The IP_1 concentrations are calculated from the difference between the experimental condition and mock transfected cells and represent biological triplicates of experimental duplicates. The values are presented as the mean and SEM.

Figure S6. Analysis of PLCγ1 variants using lipid microarrays.

(a) The principle of the assays based on LiMA are illustrated. The binding of the PLCγ1 variants (with the YFP tag) on the lipids is determined by incubation of purified proteins with the array containing different lipid compositions (i). The PLC activity of the PLCγ1 variants is assessed under the conditions where the $PI(4,5)P_2$ substrate is present and involves a second incubation with a $PI(4,5)P_2$ reporter (GFP-tagged PH domain of PLCδ) (ii).

(b) PLC activity of the tested PLCγ1 variants was probed by a consecutive incubation of the LiMA array with the PH domain of PLCγ as a reporter for $PI(4,5)P_2$. Activity is shown as the reciprocal of the NBI of PLCγ1PH on a lipid spot containing 10 mol% $PI(4,5)P_2$ and 10 mol% PS in PC as the carrier lipid. Activity of the variants is shown relative to the WT. The mean and SD of at least 3 replicates is shown.

Figure S7. XL-MS analysis of the PLCγ1/FGFRint complex.

(a) A summary of all crosslinks in the PLCγ1/FGFR1int complex showing intra-molecular crosslinks in purple and inter-molecular crosslinks in green.

(b) A subset of crosslinks showing the target residues (K, S, T or Y) in the amino acid sequences from the indicated regions of FGFR1 and PLCγ1. The intermolecular crosslinks are shown as green arrows. The intramolecular crosslinks in PLCγ1 are shown in purple. Crosslinks include residues in the β2-β3 linker/β3 region of FGFR1 kinase (including K504, T509 and K510) and residues in nSH2 (K554), nSH2-cSH2 linker (K666), cSH2 (K763) and a large portion of the spPH-nSH2 loop (including the segment K536-K549) of PLCγ1. Two PLCγ1 regions, the spPH-nSH2 and cSH2-nSH2 linkers, are in the proximity of each other and solvent exposed. The portion of the cSH2-SH3 loop, proximal to the cSH2, is similarly exposed. Distances measured in the PDB 3GQI structure between αC atoms are indicated.

Figure S8. HDX-MS analysis of the PLCγ1/FGFRint complex.

(a) Domain organisation of FGFR1int and PLCγ1 (top) and fractional deuterium uptake (bottom) for FGFR1int and PLCγ1 proteins. The width of the coloured lines

indicates the length of the peptides and the vertical grey lines indicate regions that are not covered.
(b) Difference in deuterium uptake between FGFR1int and PLCγ1 as free proteins and in the FGFR1int/ PLCγ1 complex. The grey band indicates the significance threshold.
(c) The peptides with the decreased HDX are indicated in the structure of FGFRint (PDB 3GQI) and the nSH2 domain from PLCγ1 (PDB 4FBN).

SUPPLEMENTARY REFERENCES

1. Vaque JP, Gomez-Lopez G, Monsalvez V, Varela I, Martinez N, Perez C, et al. PLCG1 mutations in cutaneous T-cell lymphomas. Blood. 2014;123(13):2034-43.
2. Caumont C, Gros A, Boucher C, Melard P, Prochazkova-Carlotti M, Laharanne E, et al. PLCG1 Gene Mutations Are Uncommon in Cutaneous T-Cell Lymphomas. The Journal of investigative dermatology. 2015;135(9):2334-7.
3. McGirt LY, Jia P, Baerenwald DA, Duszynski RJ, Dahlman KB, Zic JA, et al. Whole-genome sequencing reveals oncogenic mutations in mycosis fungoides. Blood. 2015;126(4):508-19.
4. Choi J, Goh G, Walradt T, Hong BS, Bunick CG, Chen K, et al. Genomic landscape of cutaneous T cell lymphoma. Nature genetics. 2015;47(9):1011-9.
5. Wang L, Ni X, Covington KR, Yang BY, Shiu J, Zhang X, et al. Genomic profiling of Sezary syndrome identifies alterations of key T cell signaling and differentiation genes. Nature genetics. 2015;47(12):1426-34.
6. da Silva Almeida AC, Abate F, Khiabanian H, Martinez-Escala E, Guitart J, Tensen CP, et al. The mutational landscape of cutaneous T cell lymphoma and Sezary syndrome. Nature genetics. 2015;47(12):1465-70.
7. Woollard WJ, Pullabhatla V, Lorenc A, Patel VM, Butler RM, Bayega A, et al. Candidate driver genes in Sezary syndrome: frequent perturbations of genes involved in genome maintenance and DNA repair. Blood. 2016.
8. Manso R, Rodriguez-Pinilla SM, Gonzalez-Rincon J, Gomez S, Monsalvo S, Llamas P, et al. Recurrent presence of the PLCG1 S345F mutation in nodal peripheral T-cell lymphomas. Haematologica. 2015;100(1):e25-7.
9. Yoo HY, Sung MK, Lee SH, Kim S, Lee H, Park S, et al. A recurrent inactivating mutation in RHOA GTPase in angioimmunoblastic T cell lymphoma. Nature genetics. 2014;46(4):371-5.
10. Kataoka K, Nagata Y, Kitanaka A, Shiraishi Y, Shimamura T, Yasunaga J, et al. Integrated molecular analysis of adult T cell leukemia/lymphoma. Nature genetics. 2015;47(11):1304-15.
11. Huang SC, Zhang L, Sung YS, Chen CL, Kao YC, Agaram NP, et al. Recurrent CIC Gene Abnormalities in Angiosarcomas: A Molecular Study of 120 Cases With Concurrent Investigation of PLCG1, KDR, MYC, and FLT4 Gene Alterations. Am J Surg Pathol. 2016;40(5):645-55.
12. Behjati S, Tarpey PS, Sheldon H, Martincorena I, Van Loo P, Gundem G, et al. Recurrent PTPRB and PLCG1 mutations in angiosarcoma. Nature genetics. 2014;46(4):376-9.
13. Ungewickell A, Bhaduri A, Rios E, Reuter J, Lee CS, Mah A, et al. Genomic analysis of mycosis fungoides and Sezary syndrome identifies recurrent alterations in TNFR2. Nature genetics. 2015;47(9):1056-60.
14. Kiel MJ, Sahasrabuddhe AA, Rolland DCM, Velusamy T, Chung F, Schaller M, et al. Genomic analyses reveal recurrent mutations in epigenetic modifiers and the JAK-STAT pathway in Sezary syndrome. Nat Commun. 2015;6:8470.

15. Prasad A, Rabionet R, Espinet B, Zapata L, Puiggros A, Melero C, et al. Identification of Gene Mutations and Fusion Genes in Patients with Sezary Syndrome. The Journal of investigative dermatology. 2016;136(7):1490-9.
16. Vallois D, Dobay MP, Morin RD, Lemonnier F, Missiaglia E, Juilland M, et al. Activating mutations in genes related to TCR signaling in angioimmunoblastic and other follicular helper T-cell-derived lymphomas. Blood. 2016;128(11):1490-502.
17. Manso R, Gonzalez-Rincon J, Rodriguez-Justo M, Roncador G, Gomez S, Sanchez-Beato M, et al. Overlap at the molecular and immunohistochemical levels between angioimmunoblastic T-cell lymphoma and a subgroup of peripheral T-cell lymphomas without specific morphological features. Oncotarget. 2018;9(22):16124-33.
18. Wang M, Zhang S, Chuang SS, Ashton-Key M, Ochoa E, Bolli N, et al. Angioimmunoblastic T cell lymphoma: novel molecular insights by mutation profiling. Oncotarget. 2017;8(11):17763-70.
19. McKinney M, Moffitt AB, Gaulard P, Travert M, De Leval L, Nicolae A, et al. The Genetic Basis of Hepatosplenic T-cell Lymphoma. Cancer Discov. 2017;7(4):369-79.
20. Kiel MJ, Velusamy T, Rolland D, Sahasrabuddhe AA, Chung F, Bailey NG, et al. Integrated genomic sequencing reveals mutational landscape of T-cell prolymphocytic leukemia. Blood. 2014;124(9):1460-72.
21. Kunze K, Spieker T, Gamerdinger U, Nau K, Berger J, Dreyer T, et al. A recurrent activating PLCG1 mutation in cardiac angiosarcomas increases apoptosis resistance and invasiveness of endothelial cells. Cancer Res. 2014;74(21):6173-83.
22. Murali R, Chandramohan R, Moller I, Scholz SL, Berger M, Huberman K, et al. Targeted massively parallel sequencing of angiosarcomas reveals frequent activation of the mitogen activated protein kinase pathway. Oncotarget. 2015;6(34):36041-52.
23. Ahn IE, Underbayev C, Albitar A, Herman SE, Tian X, Maric I, et al. Clonal evolution leading to ibrutinib resistance in chronic lymphocytic leukemia. Blood. 2017;129(11):1469-79.
24. Albitar A, Ma W, DeDios I, Estella J, Ahn I, Farooqui M, et al. Using high-sensitivity sequencing for the detection of mutations in BTK and PLCgamma2 genes in cellular and cell-free DNA and correlation with progression in patients treated with BTK inhibitors. Oncotarget. 2017;8(11):17936-44.
25. Burger JA, Landau DA, Taylor-Weiner A, Bozic I, Zhang H, Sarosiek K, et al. Clonal evolution in patients with chronic lymphocytic leukaemia developing resistance to BTK inhibition. Nat Commun. 2016;7:11589.
26. Gbadegesin RA, Adeyemo A, Webb NJ, Greenbaum LA, Abeyagunawardena A, Thalgahagoda S, et al. HLA-DQA1 and PLCG2 Are Candidate Risk Loci for Childhood-Onset Steroid-Sensitive Nephrotic Syndrome. J Am Soc Nephrol. 2015;26(7):1701-10.
27. Landau DA, Sun C, Rosebrock D, Herman SEM, Fein J, Sivina M, et al. The evolutionary landscape of chronic lymphocytic leukemia treated with ibrutinib targeted therapy. Nat Commun. 2017;8(1):2185.
28. Maddocks KJ, Ruppert AS, Lozanski G, Heerema NA, Zhao W, Abruzzo L, et al. Etiology of Ibrutinib Therapy Discontinuation and Outcomes in Patients With Chronic Lymphocytic Leukemia. JAMA Oncol. 2015;1(1):80-7.
29. Neves JF, Doffinger R, Barcena-Morales G, Martins C, Papapietro O, Plagnol V, et al. Novel PLCG2 Mutation in a Patient With APLAID and Cutis Laxa. Front Immunol. 2018;9:2863.
30. Woyach JA, Furman RR, Liu TM, Ozer HG, Zapatka M, Ruppert AS, et al. Resistance mechanisms for the Bruton's tyrosine kinase inhibitor ibrutinib. The New England journal of medicine. 2014;370(24):2286-94.
31. Zhou Q, Lee GS, Brady J, Datta S, Katan M, Sheikh A, et al. A hypermorphic missense mutation in PLCG2, encoding phospholipase Cgamma2, causes a

dominantly inherited autoinflammatory disease with immunodeficiency. American journal of human genetics. 2012;91(4):713-20.
32. Jones D, Woyach JA, Zhao W, Caruthers S, Tu H, Coleman J, et al. PLCG2 C2 domain mutations co-occur with BTK and PLCG2 resistance mutations in chronic lymphocytic leukemia undergoing ibrutinib treatment. Leukemia. 2017;31(7):1645-7.
33. Woyach JA, Ruppert AS, Guinn D, Lehman A, Blachly JS, Lozanski A, et al. BTK(C481S)-Mediated Resistance to Ibrutinib in Chronic Lymphocytic Leukemia. Journal of clinical oncology : official journal of the American Society of Clinical Oncology. 2017;35(13):1437-43.
34. de Lange KM, Moutsianas L, Lee JC, Lamb CA, Luo Y, Kennedy NA, et al. Genome-wide association study implicates immune activation of multiple integrin genes in inflammatory bowel disease. Nature genetics. 2017;49(2):256-61.
35. Sims R, van der Lee SJ, Naj AC, Bellenguez C, Badarinarayan N, Jakobsdottir J, et al. Rare coding variants in PLCG2, ABI3, and TREM2 implicate microglial-mediated innate immunity in Alzheimer's disease. Nature genetics. 2017;49(9):1373-84.
36. Gango A, Alpar D, Galik B, Marosvari D, Kiss R, Fesus V, et al. Dissection of subclonal evolution by temporal mutation profiling in chronic lymphocytic leukemia patients treated with ibrutinib. Int J Cancer. 2019.
37. van der Lee SJ, Conway OJ, Jansen I, Carrasquillo MM, Kleineidam L, van den Akker E, et al. A nonsynonymous mutation in PLCG2 reduces the risk of Alzheimer's disease, dementia with Lewy bodies and frontotemporal dementia, and increases the likelihood of longevity. Acta Neuropathol. 2019;138(2):237-50.
38. Read J, Rozmus J, Hannibal M. PLCG2 gene calcium-binding C2 domain variant results in autoinflammation and phospholipase C-gamma-2-associated antibody deficiency and immune dysregulation phenotype. J Clin Immunol. 2018;38:394.
